# Supplementary material for: The glucose-dependent insulinotropic polypeptide (GIP) regulates body weight and food intake via CNS-GIPR signaling
Source: Cell Metab. 2021 Apr 6;33(4):833–844.e5. doi: 10.1016/j.cmet.2021.01.015 (PMC8035082; doi:10.1016/j.cmet.2021.01.015)
Supplement: Document S1. Figures S1–S6 and Table S1 [file mmc1.pdf]

Cell Metabolism, Volume *33*

Supplemental information

The glucose-dependent insulinotropic polypeptide (GIP) regulates body weight and food intake via CNS-GIPR signaling

Qian Zhang, Challa Tenagne Delessa, Robert Augustin, Mostafa Bakhti, Gustav Colldén, Daniel J. Drucker, Annette Feuchtinger, Cristina Garcia Caceres, Gerald Grandl, Alexandra Harger, Stephan Herzig, Susanna Hofmann, Cassie Lynn Holleman, Martin Jastroch, Susanne Keipert, Maximilian Kleinert, Patrick J. Knerr, Konxhe Kulaj, Beata Legutko, Heiko Lickert, Xue Liu, Gerd Luippold, Dominik Lutter, Emilija Malogajski, Marta Tarquis Medina, Stephanie A. Mowery, Andreas Blutke, Diego Perez-Tilve, Ciro Salinno, Laura Sehrer, Richard D. DiMarchi, Matthias H. Tschöp, Kerstin Stemmer, Brian Finan, Christian Wolfrum, and Timo D. Müller

Suppl. Figure 1 – Expression of m*Gipr* in selected tissues (Related to Figure 1) and sequence of the designated peptides (Related to Figures 3-7).

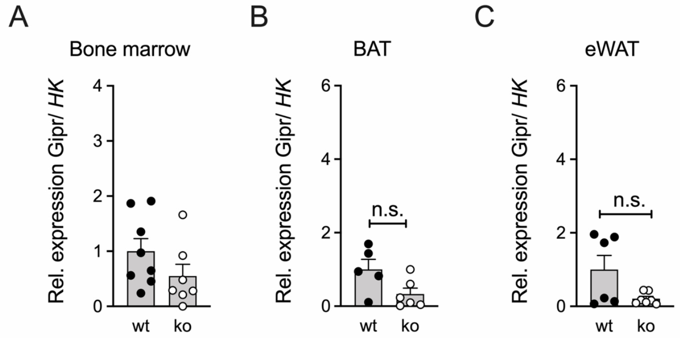

D

```
IUB 0271 (acyl-GIP)      YXEGT FISDY SIAMD KIHQQ DFVNW LLAQK GKKND WKHNI TQK (γE-γE-γE-C16)-OH
IUB 1746 (acyl-GLP-1)   HXEGT FTSDV SIYLD KQAAX EFVNW LLAGG PSSGA PPPSK(C16)
MAR709   (GLP-1/GIP)    YXEGT FTSDY SIYLD KQAAX EFVNW LLAGG PSSGA PPPSK(C16)
```

Suppl. Figure 1. Expression of m*Gipr* in selected tissues and sequence of the designated peptides. Expression of *mGipr* in bone marrow of male HFD-fed wt and CNS-*Gipr* ko mice (N=7-8 mice each group) **(a)** and in BAT **(b)** and eWAT **(c)** of male HFD-fed wt and CNS *mGipr* ko mice (N=5-7 mice each group). Peptide sequence of used molecules **(d)**.

Suppl. Figure 2 – Metabolic phenotype of Chow-fed CNS-*Gipr* ko mice (Related to Figure 1)

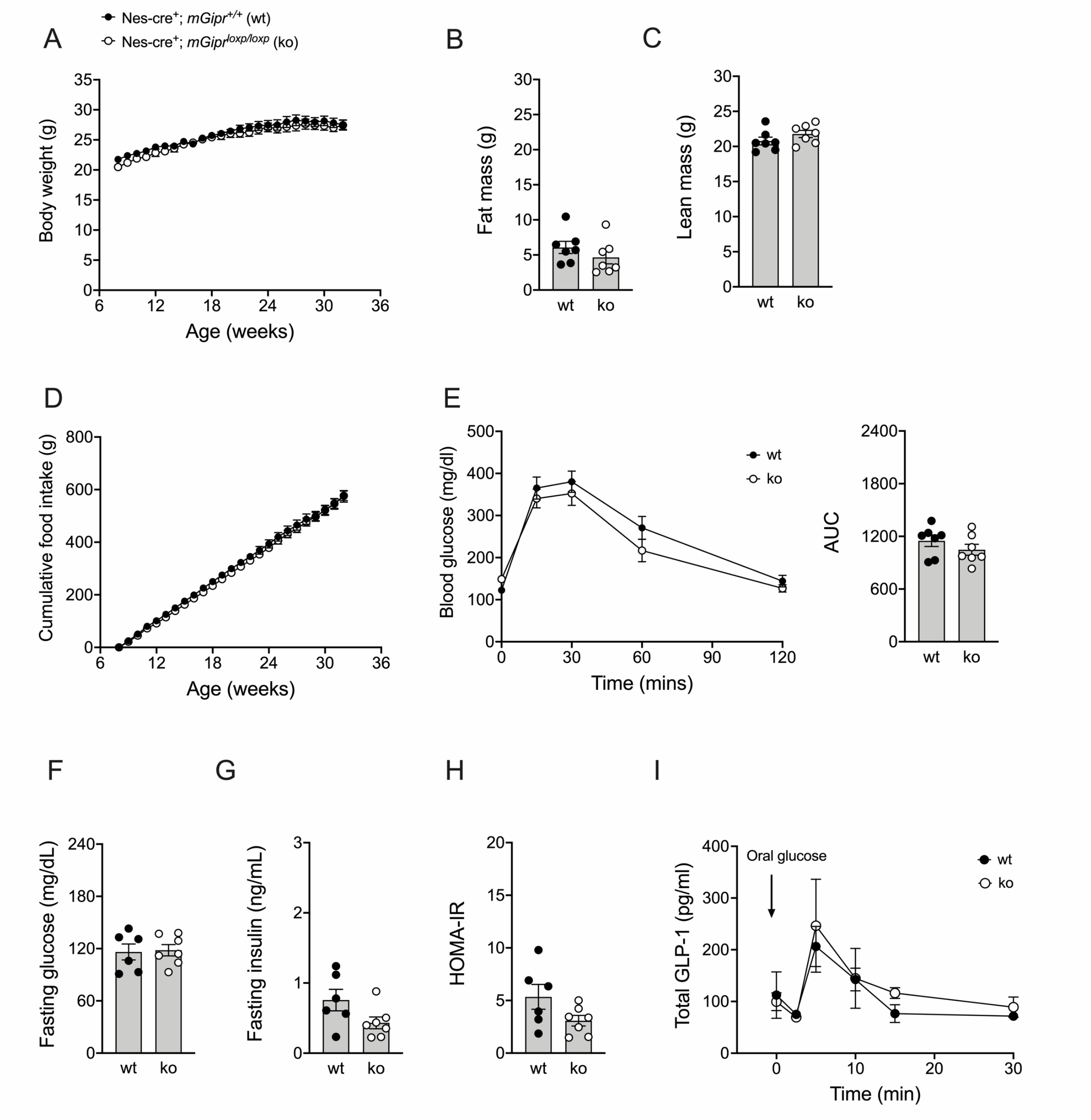

Suppl. Figure 2. Metabolic phenotype of Chow-fed CNS-*Gipr* ko mice. Body weight **(a)**, body composition at the age of 28 wks **(b,c)** and food intake **(d)** of male chow-fed C57Bl/6J wt and CNS-*Gipr* ko mice (N=7 each genotype). Food intake **(d)** was assessed per cage in double-housed mice (N=4 cages per genotype). Intraperitoneal glucose tolerance **(e)** in 28 wk old mice (N=7 each genotype) and fasting levels of blood glucose **(f)** and insulin **(g)** as well as HOMA-IR **(h)** in 32 wk old mice (N=6-7 each genotype). Oral glucose stimulation of GLP-1 secretion in 30 wk old mice (N=5-7 mice each genotype) **(i)**. Data represent means ± SEM. Asterisks indicate * $p<0.05$, ** $p<0.01$ and *** $p<0.001$. Longitudinal data **(a,d,e,i)** were analyzed using 2-way ANOVA with time and genotype as co-variables and Bonferroni post-hoc analysis for individual time-points. Bar graphs in panel (**b,c,e-h)** were analyzed using 2-tailed, 2-sided ttest.

Suppl. Figure 3 – Expression of *hGIPR* and assessment of body weight and blood glucose in *hGIPR* ko mice and different wt cohorts (Related to Figure 2).

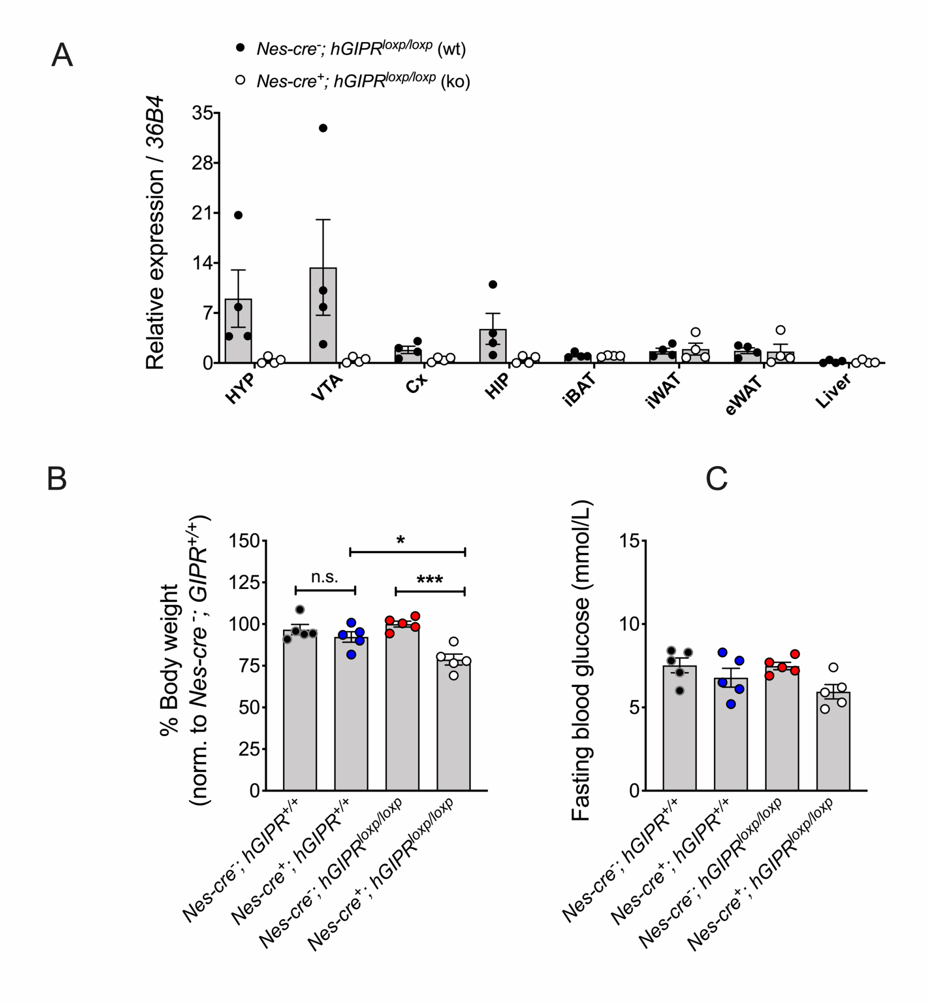

Suppl. Figure 3. GIPR ablation in CNS-*hGIPR* ko mice and assessment of body weight and blood glucose in different wt cohorts. Expression of h*GIPR* in hypothalamus, ventral tegmental area, cortex, hippocampus, inguinal brown adipose tissue (BAT), inguinal white adipose tissue (iBAT), epididymal white adipose tissue (eWAT) and liver of 12 wk old wt and CNS-*hGIPR* ko mice (N=4 each genotype) **(a)**. Difference in body weight **(b)** and fasting blood glucose **(c)** between nestin cre$^{-/-}$ *hGIPR*$^{+/+}$ mice, nestin cre$^{+/-}$ *hGIPR*$^{+/+}$ mice, nestin cre$^{-/-}$ *hGIPR*$^{flx/flx}$ mice and nestin cre$^{+/-}$ *hGIPR*$^{flx/flx}$ mice at the age of 12 wks (N=5 mice each genotype). Data represent means ± SEM. Asterisks indicate * p<0.05, ** p<0.01 and *** p<0.001. Bar graphs in (**a)** were analyzed using 2-tailed, 2-sided ttest, bar graphs in **(b,c)** were analyzed using 1-way ANOVA.

Suppl. Figure 4 – Central acyl-GIP effects on cFOS neuronal activity in the hypothalamus (Related to Figure 3).

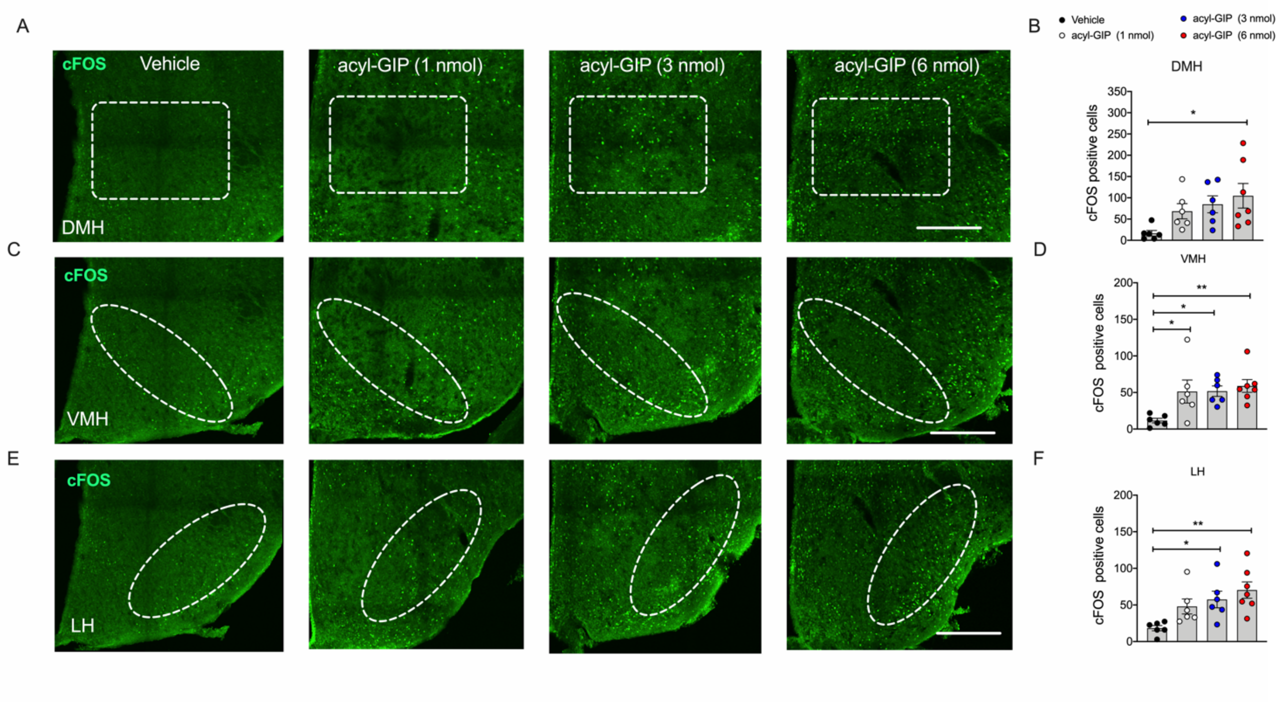

Suppl. Figure 4. Acyl-GIP effects on cFOS neuronal activity in the hypothalamus. cFOS neuronal activity in the dorsomedial nucleus **(a,b)**, the ventromedial nucleus **(c,d)** and lateral hypothalamus **(e,f)** following single acute icv treatment of DIO mice with 1, 3, or 6 nmol of acyl-GIP (N=6-7 mice each group). Data represent means ± SEM. Asterisks indicate * p<0.05, ** p<0.01 and *** p<0.001. Bar graphs in **(b,d,f)** were analyzed using 1-way ANOVA.

Suppl. Figure 5 – Effects of icv administered acyl-GIP on hypothalamic gene expression and lipid metabolism in DIO mice (Related to Figure 4).

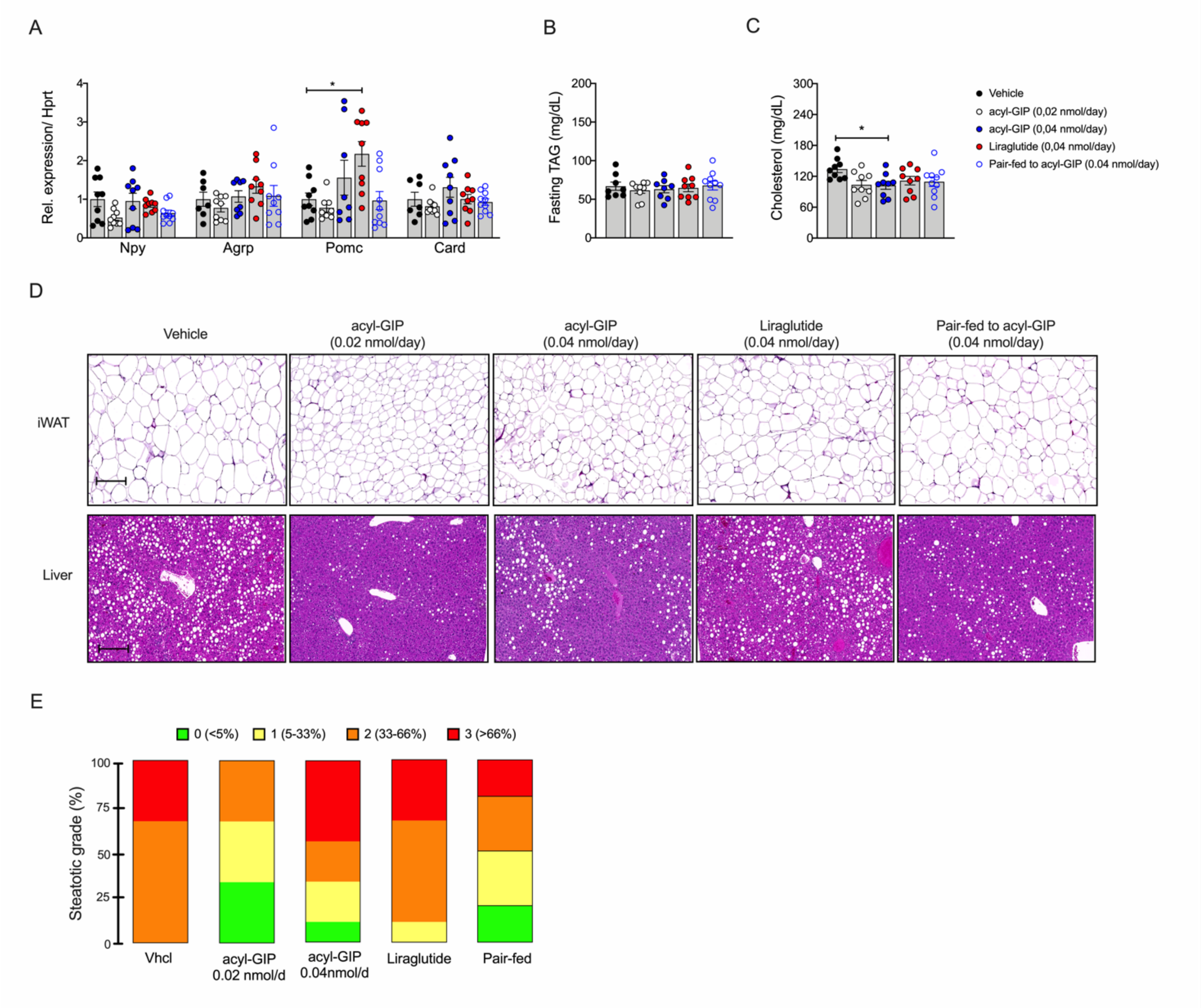

Suppl. Figure 5. Effects of icv administered acyl-GIP on hypothalamic gene expression and lipid metabolism in DIO mice. Effects of icv administered acyl-GIP (0.02 nmol/d) on hypothalamic expression of *neuropeptide y (Npy)*, *agouti-related protein (Agrp), proopiomelanocortin (Pomc) and cocaine- and amphetamine-regulated transcript (Cart)* in 32 wk old male DIO mice after 14 days of treatment (N=7-10 each group) **(a)**. Plasma levels of triglycerides **(b)** and cholesterol **(c)** after 14 days of treatment (N=8-10 mice each group). H&E staining of inguinal white adipose tissue (iWAT) and liver (scale bar 200 μm) (**d**) and quantification of the hepatic steatosis grade after 14 days of treatment (N=9-10 mice each group). Data represent means ± SEM. Asterisks indicate * p<0.05, ** p<0.01 and *** p<0.001. Bar graphs in **(a,b,c)** were analyzed using 1-way ANOVA.

Suppl. Figure 6 – Effects of acyl-GIP on thermogenic genes and BAT function (Related to Figure 5).

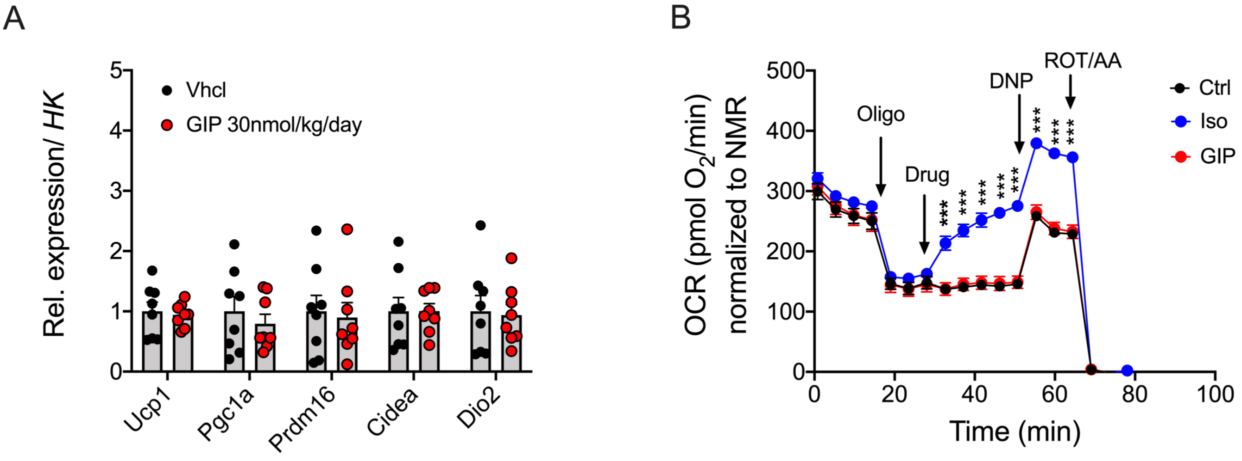

Suppl. Figure 6. Effects of acyl-GIP on thermogenic genes and BAT function. Expression of *Ucp1*, *Pgc1a*, *Prdm16*, *Cidea* and *Dio2* in BAT of male HFD-fed DIO mice treated daily with acyl-GIP (30 nmol/kg/day) for 7 days (N=8 mice each group) **(a).** Oxygen consumption rate (OCR) in individually differentiated (SV40 immortalized) brown adipocytes treated with oligomycin (5 µg/ml), test drug (isoproterenol,1 µM or GIP, 100 nM), dinitrophenol (DNP; 150 µM), followed by rotenone (5 µM) / antimycin A (2 µM) and 2DG (100 mM) **(b).** Drug effects were assessed in individually differentiated wells with N=6 Cntrl., N=3 isoproterenol, N=7 GIP. Data represent means ± SEM. Asterisks indicate * p<0.05, ** p<0.01 and *** p<0.001. Bar graphs in **(a)** were analyzed using 2-sided, 2-tailed ttest. Longitudinal data in (b) were analyzed using 2-way ANOVA with time and genotype as co-variables and Bonferroni post-hoc analysis for individual time-points.

Supplementary Table S1 – Additional Key Resource Data for used Oligonucleotides (Related to STAR Methods and Figures 1, 2, 4, Suppl. Figure 1, Suppl. Figure 3, Suppl. Figure 5, Suppl. Figure 6).

REAGENT or RESOURCE	SOURCE	IDENTIFIER
Oligonucleotides		
Pomc - F: 5’-CATTAGGCTTGGAGCAGGTC-3’	This paper	n/a
Pomc - R: 5’-TCTTGATGATGGCGTTCTTG-3’	This paper	n/a
Npy – F: 5’-TGGACTGACCCTCGCTCTAT-3’	This paper	n/a
Npy – R: 5’- TGTCTCAGGGCTGGATCTCT -3’	This paper	n/a
Agrp – F: 5’-CGGCCACGAACCTCTGTAG-3’	This paper	n/a
Agrp – R: 5’- CTCATCCCCTGCCTTTGC -3’	This paper	n/a
Cart – F: 5’-CGAGAAGAAGTACGGCCAAG-3’	This paper	n/a
Cart – R: 5’- GGAATATGGGAACCGAAGGT -3’	This paper	n/a
Ppib – F: 5’-GCATCTATGGTGAGCGCTTC-3’	This paper	n/a
Ppib – R: 5’- CTCCACCTTCCGTACCACAT -3’	This paper	n/a
Hprt – F: 5’-AAGCTTGCTGGTGAAAAGGA-3’	This paper	n/a
Hprt – R: 5’- TTGCGCTCATCTTAGGCTTT -3’	This paper	n/a
Gipr universal – F: 5’-CCAGGAGTGCCAAAAGATGTT-3’	This paper	n/a
Gipr universal – R: 5’-CCAGGAGTGCCAAAAGATGTT-3’	This paper	n/a
Gipr wt-specific – F: 5’-GTGTCCACGAGGTGGTGTTT -3’	This paper	n/a
Gipr universal – R: 5’-CCGACTGCACCTCTTTGTTG -3’	This paper	n/a
hGIPR– F: 5’-TGTTTGCTCCCGTGACAGAG -3’	This paper	n/a
hGIPR – R: 5’-CGACTGCACCTCCTTGTTGA -3’	This paper	n/a
Pomc– F: 5’-ACCCTCGTTTCTCTGCGCATC -3’	This paper	n/a
Pomc– R: 5’-AGGGCCCCTGAGCGACTGTA -3’	This paper	n/a
Npy– F: 5’- TGGACTGACCCTCGCTCTAT -3’	This paper	n/a
Npy– R: 5’- GGGGCGTTTTCTGTGCTTTC -3’	This paper	n/a
AgRP– F: 5’- GCCATCTTCCACCTTTGCAG -3’	This paper	n/a
AgRP– R: 5’- CCTGGTCAGGCCTTCTGATG -3’	This paper	n/a
Cart– F: 5’- CCTGCTGCTACTGCTACCTTT -3’	This paper	n/a
Cart– R: 5’- CGCTTCGATCAGCTCCTTCT -3’	This paper	n/a
Bdnf– F: 5’- CATCCGAGGACAAGGTGGCTTG -3’	This paper	n/a
Bdnf– R: 5’- GCCGAACTTTCTGGTCCTCATC -3’	This paper	n/a
36B4– F: 5’- GCCGTGATGCCCAGGGAAGACA -3’	This paper	n/a
36B4– R: 5’- AACCCGCGGCCTTCTACGAG -3’	This paper	n/a
Ucp1– F: 5’- GGCCTCTACGACTCAGTCCA-3’	This paper	n/a
Ucp1– R: 5’-TAAGCCGGCTGAGATCTTGT-3’	This paper	n/a
Prdm16– F: 5’- CCGCTGTGATGAGTGTGATG-3’	This paper	n/a
Prdm16– R: 5’- GGACGATCATGTGTTGCTCC-3’	This paper	n/a
Cidea– F: 5’- AATGGACACCGGGTAGTAAGT-3’	This paper	n/a
Cidea– R: 5’- CAGCCTGTATAGGTCGAAGGT-3’	This paper	n/a
Dio2– F: 5’- TGCCACCTTCTTGACTTTGC-3’	This paper	n/a
Dio2– R: 5’- GGTTCCGGTGCTTCTTAACC-3’	This paper	n/a
Pgc1a– F: 5’- AGCCGTGACCACTGACAACGAG-3’	This paper	n/a
Pgc1a– R: 5’- GCTGCATGGTTCTGAGTGCTAAG -3’	This paper	n/a
