# Supplementary material for: The glucose-dependent insulinotropic polypeptide (GIP) regulates body weight and food intake via CNS-GIPR signaling
Source: Cell Metab. 2021 Apr 6;33(4):833–844.e5. doi: 10.1016/j.cmet.2021.01.015 (PMC8035082; doi:10.1016/j.cmet.2021.01.015)
Supplement: Document S2. Article plus supplemental information [file mmc2.pdf]

Cell Metabolism

Article

The glucose-dependent insulinotropic polypeptide (GIP) regulates body weight and food intake via CNS-GIPR signaling

Graphical Abstract

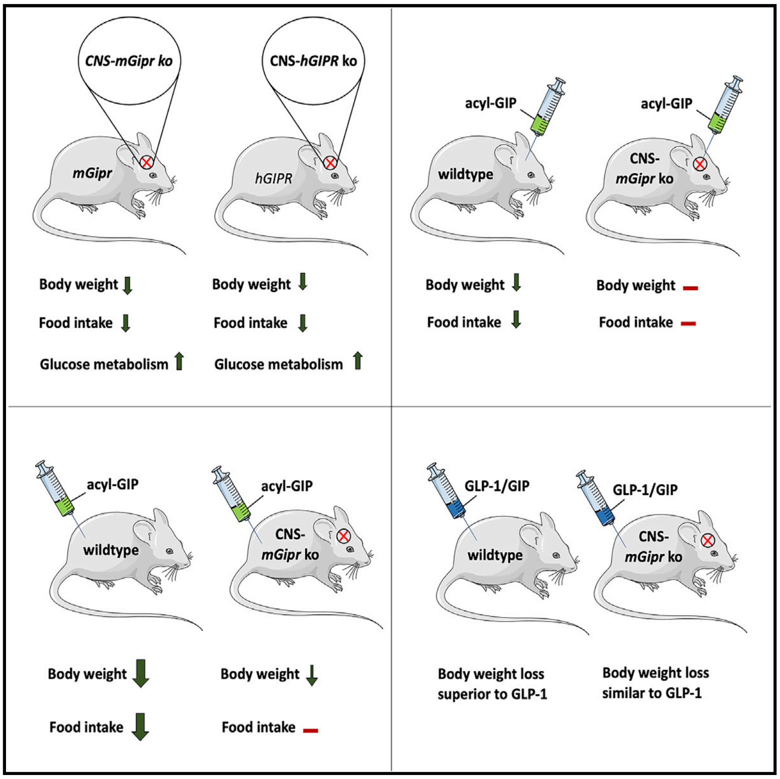

Highlights

- CNS-*Gipr* KO mice are protected from diet-induced obesity and glucose intolerance
- Acyl-GIP increases cFOS neuronal activity in key hypothalamic feeding centers
- Acyl-GIP effects on body weight and food intake are absent/ blunted in *CNS-mGipr* KO mice
- GLP-1/GIP dual-agonism loses superior potency over GLP-1 in *CNS-mGipr* KO mice.

Authors

Qian Zhang, Challa Tenagne Delessa, Robert Augustin, ..., Brian Finan, Christian Wolfrum, Timo D. Müller

Correspondence

timo.mueller@helmholtz-muenchen.de

In Brief

Zhang et al. report that CNS GIPR plays a significant role in regulating food intake. They show that treatment with acyl-GIP or with a GLP-1/GIP dual-agonist lowers body weight and food intake in wild-type mice but shows blunted efficacy in CNS-Gipr KO mice.

Zhang et al., 2021, Cell Metabolism *33*, 833–844
April 6, 2021 © 2021 The Authors. Published by Elsevier Inc.
https://doi.org/10.1016/j.cmet.2021.01.015

CellPress

Cell Metabolism

CellPress
OPEN ACCESS

Article

The glucose-dependent insulinotropic polypeptide (GIP) regulates body weight and food intake via CNS-GIPR signaling

Qian Zhang,[1,2] Challa Tenagne Delessa,[3] Robert Augustin,[4] Mostafa Bakhti,[2,5] Gustav Colldén,[1,2] Daniel J. Drucker,[6] Annette Feuchtinger,[7] Cristina Garcia Caceres,[1,2] Gerald Grandl,[1,2] Alexandra Harger,[1,2] Stephan Herzig,[2,8,9] Susanna Hofmann,[2,5,10] Cassie Lynn Holleman,[1,2] Martin Jastroch,[11] Susanne Keipert,[11] Maximilian Kleinert,[1,2] Patrick J. Knerr,[12] Konxhe Kulaj,[1,2] Beata Legutko,[1,2] Heiko Lickert,[2,5,13] Xue Liu,[1,2] Gerd Luippold,[4] Dominik Lutter,[1,2] Emilija Malogajski,[1,2] Marta Tarquis Medina,[2,5,13] Stephanie A. Mowery,[12] Andreas Blutke,[7] Diego Perez-Tilve,[14] Ciro Salinno,[2,5,13] Laura Sehrer,[1,2] Richard D. DiMarchi,[15] Matthias H. Tschöp,[2,16,17] Kerstin Stemmer,[1,2] Brian Finan,[12] Christian Wolfrum,[3] and Timo D. Müller[1,2,18,19,*]

[1]Institute for Diabetes and Obesity, Helmholtz Diabetes Center, Helmholtz Zentrum München, Neuherberg, Germany
[2]German Center for Diabetes Research (DZD), Neuherberg, Germany
[3]Institute of Food, Nutrition and Health, Department of Health Sciences and Technology (D-HEST), ETH Zürich, Zurich, Switzerland
[4]Cardiometabolic Diseases Research Department, Boehringer Ingelheim Pharma GmbH and Co., KG, Biberach/Riss, Germany
[5]Institute of Diabetes and Regeneration Research, Helmholtz Zentrum München, 85764 Neuherberg, Germany
[6]Lunenfeld-Tanenbaum Research Institute, Mt. Sinai Hospital, University of Toronto, Toronto, ON M5G 1X5, Canada
[7]Research Unit Analytical Pathology, Helmholtz Zentrum München, Neuherberg, Germany
[8]Institute for Diabetes and Cancer, Helmholtz Diabetes Center, Helmholtz Center Munich, Neuherberg, Germany
[9]Molecular Metabolic Control, Technical University of Munich, Munich, Germany
[10]Medizinische Klinik und Poliklinik IV, Klinikum der LMU, München, Germany
[11]Department of Molecular Biosciences, The Wenner-Gren Institute, The Arrhenius Laboratories F3, Stockholm University, Stockholm, Sweden
[12]Novo Nordisk Research Center Indianapolis, Indianapolis, IN 46241, USA
[13]Technische Universität München, School of Medicine, Klinikum Rechts der Isar, 81675 München, Germany
[14]Department of Pharmacology and Systems Physiology, University of Cincinnati College of Medicine, Cincinnati, OH, USA
[15]Department of Chemistry, Indiana University, Bloomington, IN 47405, USA
[16]Helmholtz Zentrum München, Neuherberg, Germany
[17]Technische Universität München, München, Germany
[18]Department of Pharmacology and Experimental Therapy, Institute of Experimental and Clinical Pharmacology and Toxicology, Eberhard Karls University Hospitals and Clinics, 72076 Tübingen, Germany
[19]Lead contact
*Correspondence: timo.mueller@helmholtz-muenchen.de
https://doi.org/10.1016/j.cmet.2021.01.015

SUMMARY

Uncertainty exists as to whether the glucose-dependent insulinotropic polypeptide receptor (GIPR) should be activated or inhibited for the treatment of obesity. Gipr was recently demonstrated in hypothalamic feeding centers, but the physiological relevance of CNS Gipr remains unknown. Here we show that HFD-fed CNS-*Gipr* KO mice and humanized *(h)GIPR* knockin mice with CNS-*hGIPR* deletion show decreased body weight and improved glucose metabolism. In DIO mice, acute central and peripheral administration of acyl-GIP increases cFos neuronal activity in hypothalamic feeding centers, and this coincides with decreased body weight and food intake and improved glucose handling. Chronic central and peripheral administration of acyl-GIP lowers body weight and food intake in wild-type mice, but shows blunted/absent efficacy in CNS-*Gipr* KO mice. Also, the superior metabolic effect of GLP-1/GIP co-agonism relative to GLP-1 is extinguished in CNS-*Gipr* KO mice. Our data hence establish a key role of CNS Gipr for control of energy metabolism.

INTRODUCTION

The glucose-dependent insulinotropic polypeptide (GIP) regulates blood glucose via its insulinotropic and glucagonotropic action on the pancreas (Christensen et al., 2011; Finan et al., 2016). While the glycemic effects of GIP receptor (GIPR) agonism are solidly confirmed, uncertainty exists as to whether GIPR should be stimulated or inhibited for the treatment of

This is an open access article under the CC BY license (http://creativecommons.org/licenses/by/4.0/).

type 2 diabetes mellitus (T2DM) and obesity (Holst and Rosenkilde, 2020). Global germline *Gipr* knockout (KO) mice show lower body weight and preserved insulin sensitivity upon high-fat diet (HFD) feeding (Miyawaki et al., 2002), and the insulinotropic response to GIP is impaired in patients with T2DM (Nauck et al., 1993). GIP activates lipoprotein lipase (Eckel et al., 1979; Kim et al., 2007, 2010), stimulates uptake of fatty acids and glucose (Beck and Max, 1986; Hauner et al., 1988), and promotes lipid synthesis in cultured adipocytes (Hauner et al., 1988). These data align with studies in humans, in which GIP is shown to promote lipid storage by increasing adipose tissue blood flow and triglyceride uptake (Asmar et al., 2017). These and other data have spurred the development of GIPR antagonists for the treatment of T2DM and obesity. Recently, it was shown that GIPR antagonizing antibodies improve body weight and glucose control in mice and non-human primates (Killion et al., 2018) and enhance the anorectic effect of leptin in HFD-fed mice (Kaneko et al., 2019). In contrast to these data, overexpression of Gip improves body weight and glycemia in HFD-fed mice (Kim et al., 2012). Pigs expressing a dominant-negative Gipr in the β cells are glucose intolerant and show reduced glucose stimulation of insulin secretion (Renner et al., 2010). Optimized GIP analogs decrease body weight in wild-type (WT) and *GLP-1 receptor* (*GLP-1R*) KO mice, but fail to do so in mice deficient for *Gipr* (Mroz et al., 2019). Co-administration of a GLP-1R agonist with a GIPR agonist synergistically decreases body weight and fat mass in diet-induced obese (DIO) mice (Finan et al., 2013). Unimolecular dual-agonists targeting the receptors for GLP-1 and GIP decrease body weight and improve glucose handling in animal models of obesity and T2DM (Coskun et al., 2018; Finan et al., 2013), non-human primates (Finan et al., 2013), and obese patients with T2DM (Coskun et al., 2018; Finan et al., 2013; Frias et al., 2018). Moreover, the dual-agonists exhibit greater efficacy relative to GLP-1R agonism alone in preclinical studies (Coskun et al., 2018; Finan et al., 2013) and clinical trials (Frias et al., 2018). In summary, there is considerable uncertainty as to how GIPR agonism versus antagonism improves energy metabolism.

Expression of *Gipr* was recently demonstrated in cells/neurons of the arcuate (ARC), dorsomedial (DMH), and paraventricular (PVH) nuclei of the hypothalamus, and Gq-DREADD-mediated activation of these neurons/cells decreases food intake in mice (Adriaenssens et al., 2019). While these data indicate that Gipr is located on hypothalamic neurons that control feeding behavior, these *Gipr*-expressing neurons/cells most likely also express other factors that affect food intake. Hence, it remains unclear whether CNS Gipr signaling is of relevance for energy metabolism control in general and for the metabolic effects of GIP-based pharmacotherapies specifically.

The aim of our studies is to assess the role of CNS Gipr in the systemic regulation of body weight, food intake, energy expenditure, and glucose metabolism. We show that mice with CNS deletion of murine *(m)Gipr* (nestin cre$^{+/-}$ *mGipr*$^{flx/fl}$) and also humanized *(h)GIPR* knockin mice with conditional CNS deletion of *hGIPR* (nestin cre$^{+/-}$ *hGIPR*$^{flx/flx}$) phenocopy global germline *Gipr* KO mice with respect to lower body weight and improved glucose metabolism upon HFD feeding. The lower body weight of CNS-*mGipr* KO mice and of CNS-*hGIPR* KO mice is accompanied by decreased food intake without changes in energy expenditure. Consistent with localization of *Gipr* in hypothalamic nuclei linked to control of appetite (Adriaenssens et al., 2019), we show that acute central and peripheral administration of fatty acyl-GIP increases cFOS neuronal activity in key hypothalamic feeding centers and that this coincides acutely and chronically with decreased body weight, food intake, and blood glucose. Chronic central (intracerebroventricular, i.c.v.) and peripheral (subcutaneous, s.c.) treatment with fatty acyl-GIP improves body weight and food intake in DIO WT mice, but this effect is absent upon i.c.v. fatty acyl-GIP treatment and blunted upon s.c. acyl-GIP treatment in CNS-*Gipr* KO mice. Also, the superior metabolic effects of unimolecular GLP-1/GIP dual-agonism relative to treatment with GLP-1 alone are extinguished in CNS-*Gipr* KO mice. In summary, our data reveal new roles for CNS Gipr signaling in control of energy metabolism and indicate that central Gipr signaling is essential for the anorectic effect of GIPR agonism and the metabolic benefits of dual GLP-1/GIP agonists.

RESULTS

Mice with CNS-specific deletion of *mGipr* are protected from diet-induced obesity and glucose intolerance

To evaluate the role of CNS Gipr signaling for systemic energy metabolism control, we generated mice in which *mGipr* is deleted in the CNS by crossing *mGipr*$^{flx/flx}$ mice (Ussher et al., 2018) with mice that express the cre recombinase under control of the nestin promoter. Consistent with the phenotype of germline global *Gipr* KO mice (Miyawaki et al., 2002), CNS-m*Gipr* KO mice showed lower body weight relative to WT control mice when chronically fed with an HFD (Figure 1A). Consistent with this, body fat, but not lean tissue mass, was decreased in CNS-m*Gipr* KO mice (Figures 1B and 1C). CNS-m*Gipr* KO mice exhibited reduced food intake (Figure 1D) with unchanged assimilated energy per gram eaten food (Figure 1E) and increased locomotor activity (Figure 1F) without transcriptional changes in hypothalamic *Npy*, *Agrp*, *Pomc*, or *Cart* (Figure 1G). No changes were observed in total energy expenditure, resting metabolic rate (Figures 1H and 1I), or expression of genes related to thermogenesis in brown adipose tissue (BAT) (Figure 1J). Also, substrate utilization (Figure 1K) and plasma levels of triglycerides and cholesterol (Figures 1L and 1M) were unchanged. CNS-m*Gipr* KO mice showed improved glucose tolerance (Figures 1N and 1O) and decreased HbA1c (Figure 1P) without differences in fasting levels of blood glucose (Figure 1Q), but lower plasma levels of insulin (Figure 1R) and improved insulin sensitivity approximated by HOMA-IR (Figure 1S). Gene expression profiling showed a robust (∼95%) decrease in *Gipr* mRNA in the hypothalamus (Figure 1T) of CNS-*Gipr* KO mice with unchanged expression in isolated islets (Figure 1U), bone marrow, or white (WAT) and brown adipose tissue (BAT) (Figures S1A–S1C). In line with preservation of *Gipr* expression in the islets (Figure 1U), we saw no difference in glucose-stimulated insulin secretion (GSIS) between islets isolated from WT and CNS-*mGipr* KO mice (Figure 1V) and preservation of GLP-1 and GIP to stimulate islet insulin secretion (Figure 1W). Collectively, these data demonstrate that islet incretin action is not compromised in the CNS-*mGipr* KO mice.

Similar to the phenotype of the global germline *Gipr* KO mice (Miyawaki et al., 2002), we saw no difference in body weight,

CellPress
OPEN ACCESS

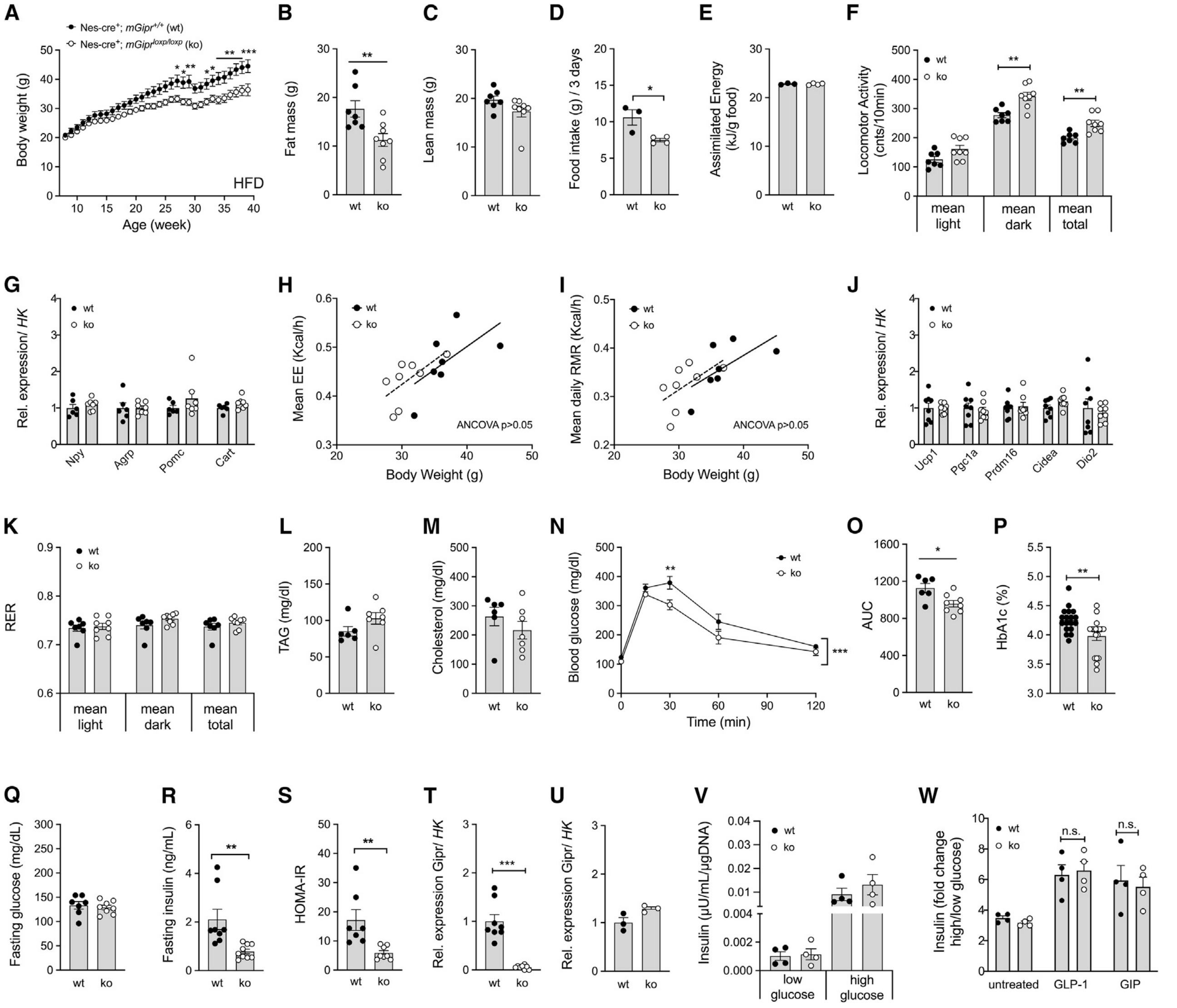

Figure 1. Mice with CNS deletion of murine *Gipr* are protected from diet-induced obesity and glucose intolerance

(A–E) Body weight (A), body composition at the age of 28 weeks (B and C), food intake (D), and assimilated energy (E) in 42-week-old male C57BL/6J WT and CNS-*Gipr* KO mice (N = 7–8 mice each group) fed with a high-fat diet (HFD). Food intake and assimilated energy were assessed per cage in double-housed mice.

(F–I) Locomotor activity (N = 7–8 mice each group) (F), hypothalamic expression of genes related to food intake (6–7 mice each group) (G), and total energy expenditure (H) and resting metabolic rate (I) in 29-week-old male mice (N = 7–8 mice each group).

(J) Expression of genes related to BAT thermogenesis in HFD-fed male mice (N = 8 each genotype).

(K) Respiratory exchange ratio (RER) in 29-week-old male mice (N = 7–8 mice each group).

(L–O) Plasma levels of triglycerides (L) and cholesterol (M) (N = 6–7 each group) and intraperitoneal glucose tolerance (N and O) (N = 6–8 mice each group) in 42-week-old male mice.

(P) HbA1c (N = 18 mice each group; p = 0.0033).

(Q and R) Fasting levels of blood glucose (Q) and insulin (R) as well as HOMA-IR (S) in 42-week-old male mice (N = 7–8 each group).

(T and U) Relative expression of *Gipr* (corrected to housekeeping gene peptidylprolyl isomerase B; *Ppib*) in the hypothalamus (N = 8 mice each genotype) (T) and in isolated islets from WT and CNS-*Gipr* KO mice (N = 3 each group) (U).

(V and W) Glucose-stimulated insulin secretion (GSIS) in isolated islets under conditions of low (2.8 mM) and high glucose (16.8 mM) (V) and GSIS of isolated islets treated with or without 10 nM of either acyl-GLP-1 or acyl-GIP (W) (N = 4 mice each group). y axis in (W) represents the ratio of secreted insulin stimulated with high glucose (16.8 mM) to low glucose (2.8 mM).

Data represent means ± SEM. *p < 0.05, **p < 0.01, and ***p < 0.001. Longitudinal data (A and N) were analyzed using two-way ANOVA with time and genotype as co-variables and Bonferroni post hoc analysis for individual time points. Bar graphs (B–G, J–M, and O–W) were analyzed using two-tailed, two-sided t test. Data in (H) and (I) were analyzed using ANCOVA with body weight as co-variate.

fat or lean tissue mass, food intake, or glucose tolerance when CNS-*mGipr* KO mice were fed with a standard chow diet (Figures S2A–S2E). Further, no differences were seen in either plasma levels of blood glucose and insulin or in insulin sensitivity approximated by HOMA-IR (Figures S2F–S2H). Also, plasma levels of GLP-1 were unchanged between WT and CNS-*mGipr* KO mice under both baseline conditions and after oral administration of glucose (Figure S2I). In summary, these data show that CNS-specific loss of m*Gipr* phenocopies the global germline loss of *Gipr* with regard to lower body weight and improved glucose metabolism under HFD, but not chow-fed conditions.

Humanized *GIPR* knockin mice with conditional CNS-specific *hGIPR* deletion are protected from diet-induced obesity and glucose intolerance

To further validate the phenotype arising from selective elimination of the CNS-*Gipr*, we generated *hGIPR* knockin mice with conditional nestin cre-mediated *hGIPR* deletion in the CNS. Such CNS-*hGIPR* KO mice showed decreased mRNA levels of *hGIPR* in the hypothalamus, ventral tegmental area (VTA), hippocampus, and cortex with unchanged *GIPR* expression in BAT, inguinal (iWAT) and epididymal WAT (eWAT), and the liver (Figure S3A). Consistent with the phenotype of the CNS-specific *mGipr* KO mice, the CNS-specific *hGIPR* KO mice also showed reduced weight gain over time upon HFD exposure relative to the humanized control mice (Figure 2A). The lower body weight of the CNS-specific *hGIPR* KO mice was paralleled by a decrease in body fat and lean tissue mass (Figures 2B and 2C) and food intake (Figure 2D) that is associated with increased expression of *Pomc*, *Bdnf*, and *Cart* in the hypothalamus (Figure 2E). Consistent with the CNS-*mGIPR* KO mice, the CNS-*hGIPR* KO mice also showed no difference in energy expenditure, but trended toward increased physical activity in the dark phase (Figures 2F and 2G). Consistent with the lower body weight, we also saw improved glucose metabolism in CNS-specific *hGIPR* KO mice, as indicated by enhanced glucose tolerance (Figure 2H) and decreased fasting levels of glucose and insulin (Figures 2I and 2J). Fasting plasma levels of total GLP-1 were increased in CNS-*hGIPR* KO mice while levels of leptin, triglycerides, and non-esterified fatty acids (NEFAs) were decreased (Figures 2K–2N). No difference was observed in plasma levels of GIP and cholesterol (Figures 2O and 2P), but hepatic lipid accumulation was decreased in CNS-*hGIPR* KO mice (Figure 2Q). In summary, mice with CNS-specific ablation of the murine or human GIP receptor phenocopy global *Gipr* KO mice with respect to reduced weight gain and improved glucose metabolism upon HFD feeding. Notably, no difference in body weight or blood glucose was observed between WT nestin cre$^{-/-}$ *hGIPR*$^{flx/flx}$, nestin cre$^{+/-}$ *hGIPR*$^{wt/wt}$, and nestin cre$^{-/-}$ *hGIPR*$^{wt/wt}$ mice (Figures S3B and S3C).

Acute central administration of fatty acyl-GIP improves body weight, food intake, and glycemia in DIO mice

We next assessed the acute metabolic effects of single i.c.v. administration of a validated long-acting (fatty acylated) GIP (IUB0271; Figure S1D) in DIO mice (Mroz et al., 2019). Acute i.c.v. administration of fatty acyl-GIP dose-dependently decreased body weight relative to vehicle controls (Figure 3A). We saw reduced food intake in fatty acyl-GIP-treated mice (Figures 3B and 3C), and this was paralleled by an acute decrease of blood glucose in all treatment groups within the first 3 h, persisting for 24 h in mice treated with 6 nmol fatty acyl-GIP (Figures 3D and 3E). No difference was observed in plasma levels of insulin, c-peptide, triglycerides, or free fatty acids (Figures 3F–3I). Consistent with the lower body weight and food intake (Figures 3A and 3B), and with recent reports demonstrating that GIPR is present in key hypothalamic feeding centers (Adriaenssens et al., 2019), we saw a dose-dependent increase in cFOS neuronal activity in the ARC (Figures 3J and 3K) as well as in nuclei of the DMH, ventromedial hypothalamus (VMH), and lateral hypothalamus (LH), following administration of acyl-GIP (Figures S4A–S4F). Collectively, these data show that centrally administered fatty acyl-GIP acutely reduces body weight, food intake, and glycemia in DIO mice, and that this correlated with increased neuronal activation (cFOS) in key feeding centers of the hypothalamus. These data reveal that pharmacological activation of CNS-GIPR signaling is relevant for energy metabolism.

Chronic central administration of fatty acyl-GIP reduced body weight, food intake, and glycemia in HFD-fed WT mice, but not in CNS-*Gipr* KO mice

We next continuously infused fatty acyl-GIP centrally (i.c.v.) at doses of either 0.02 or 0.04 nmol/day for 12 days in DIO mice and compared its metabolic effects to mice that were pair-fed to match the food intake of the fatty acyl-GIP (0.04 nmol/day)-treated mice as well as to mice treated with liraglutide (0.04 nmol/day). Both acyl-GIP-treated groups show greater body weight loss relative to mice treated with vehicle or liraglutide (Figure 4A). After 6 days of treatment, mice treated with fatty acyl-GIP (0.04 nmol/day) exhibit greater weight loss relative to the pair-fed controls (Figure 4A). Consistent with the ability of fatty acyl-GIP to decrease body weight, we saw fat mass and food intake decreased (Figures 4B and 4C), but this was not paralleled by transcriptional changes in hypothalamic *Npy*, *Agrp*, *Pomc*, or *Cart* (Figure S5A). These data thus indicate that most, but not all, of the body weight-lowering effect of centrally administered fatty acyl-GIP is mediated by inhibition of food intake. Fasting levels of blood glucose were decreased in all treatment groups, but with the greatest improvement in mice treated with the highest dose of fatty acyl-GIP (Figure 4D). Similarly, mice treated with the highest dose of fatty acyl-GIP showed lower fasting levels of insulin and leptin (Figures 4E and 4F) and improved insulin sensitivity relative to the vehicle controls (Figure 4G). Fatty acyl-GIP-mediated lowering of body weight and glycemia was not related to transcriptional changes of *Gipr* in the hypothalamus or the adipose tissue (Figure 4H). No difference was observed in plasma levels of triglycerides or cholesterol (Figures S5B and S5C), but consistent with the decreased body weight, mice treated with acyl-GIP at both dose levels showed decreased adipocyte size in the iWAT and reduced hepatostatosis (Figures S5D and S5E).

To further interrogate the role of central Gipr agonism on energy metabolism, we chronically (i.c.v.) infused fatty acyl-GIP at a dose of 0.02 nmol/day in HFD-fed WT and CNS-*Gipr* KO mice. Consistent with previous data, body weight, food intake, and blood glucose were decreased in WT mice treated with fatty acyl-GIP, but centrally administered acyl-GIP failed to improve body weight, food intake, or blood glucose in CNS-*Gipr* KO

CellPress
OPEN ACCESS

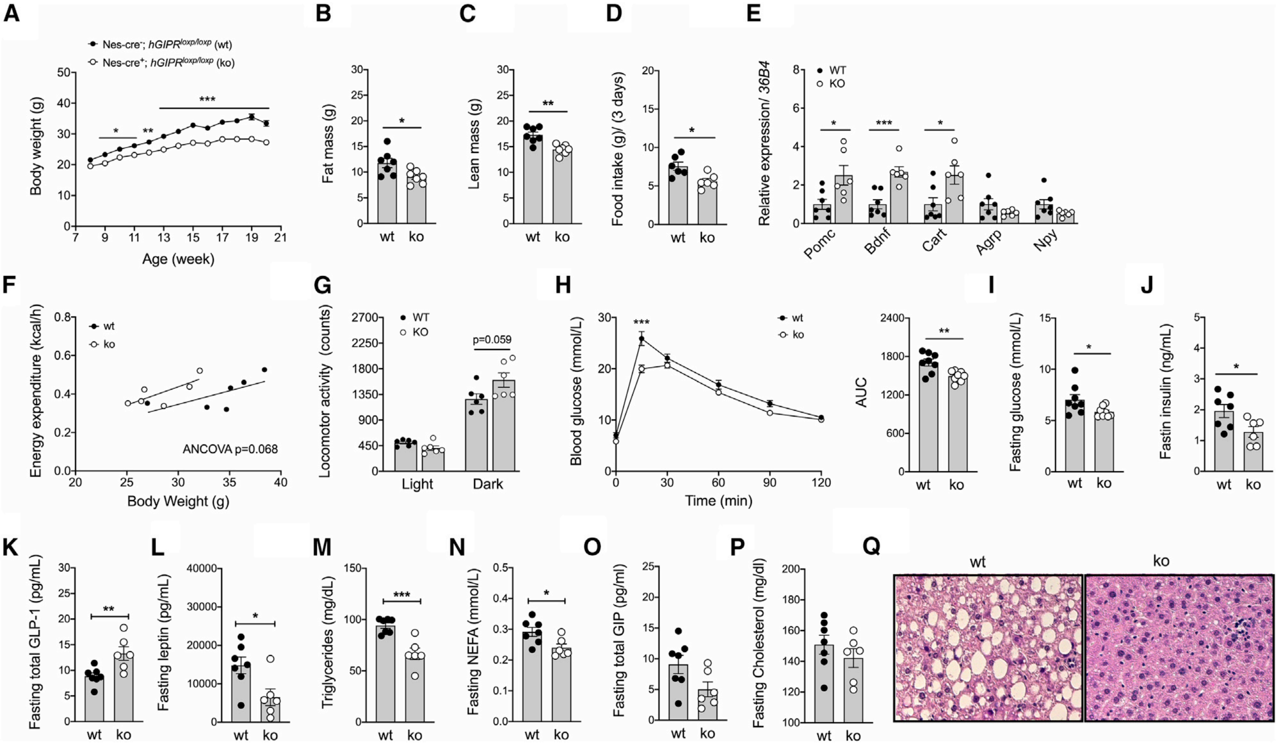

Figure 2. Humanized (h)GIPR knockin mice with conditional CNS-specific *hGIPR* deletion are protected from diet-induced obesity and glucose intolerance

(A–D) Body weight (A), body composition (B and C), and food intake (D) in male C57BL/6N WT and CNS-*hGIPR* KO mice (N = 6–8 mice each group).

(E) Hypothalamic expression of *proopiomelanocortin* (*Pomc*), *brain-derived neurotrophic factor* (*Bdnf*), *cocaine-and-amphetamine-regulated transcript* (*Cart*), *agouti-related peptide* (*Agrp*), and *neuropeptide y* (*Npy*) in 20-week-old male mice (N = 6–7 mice each group).

(F and G) Energy expenditure (F) and locomotor activity (G) in 20-week-old male mice (N = 6 mice each group).

(H–P) Intraperitoneal glucose tolerance (H) and fasting levels of blood glucose (I), insulin (J), GLP-1 (K), leptin (L), triglycerides (M), free fatty acids (N), GIP (O), and cholesterol (P) in WT and CNS-*hGIPR* KO mice (N = 6–8 mice each group).

(Q) H&E staining of hepatic lipid accumulation (scale bar represents 100 μm).

Data represent means ± SEM. *$p < 0.05$, **$p < 0.01$, and ***$p < 0.001$. Longitudinal data (A and H) were analyzed using two-way ANOVA with time and genotype as co-variables and Bonferroni post hoc analysis for individual time points. Bar graphs (B–E and G–P) were analyzed using two-tailed, two-sided t test. Data in (F) were analyzed using ANCOVA with body weight as co-variate.

mice (Figures 4I–4K). Hence, the ability of centrally administered fatty acyl-GIP to lower body weight, food intake, and glycemia requires the CNS GIPR.

Peripheral administration of fatty acyl-GIP reduces body weight through inhibition of food intake without affecting energy expenditure

We next assessed the metabolic effects of peripherally injected fatty acyl-GIP in DIO mice. Chronic peripheral (s.c.) administration of acyl-GIP decreased body weight in DIO mice (Figure 5A), and this was paralleled by both acute and sustained inhibition of food intake (Figures 5B and 5C) with a greater preference for smaller meals without difference in meal frequency (Figures 5D and 5E). Consistent with the inhibition of food intake, GIP treatment acutely increased fatty acid oxidation (Figure 5F), and this correlated with enhanced lipid utilization, as indicated by a lower respiratory exchange ratio (RER) (Figure 5G). Peripheral administration of acyl-GIP neither acutely nor chronically affected energy expenditure (Figures 5H and 5I) or genes related to thermogenesis in BAT (Figure S6A). Consistent with this, we saw no effect of GIP on oxygen consumption in cultured brown adipocytes (Figure S6B). Interestingly, however, acyl-GIP decreased assimilated energy and assimilation efficiency (Figures 5J and 5K), indicating that peripheral delivery of GIP, apart from decreasing food intake, also decreases the amount of metabolizable energy. In line with our data showing that central administration of acyl-GIP increased cFOS neuronal activity in key hypothalamic feeding centers (Figures 3J, 3K, and S4A–S4F), we also saw cFOS increased in the ARC and the VMH after peripheral GIP treatment (Figures 5L–5N). Consistent with the observation that centrally administered acyl-GIP does not change expression of *Npy* or *Pomc* (Figure S5A), we saw no differences in c-FOS/NPY co-localization after peripheral acyl-GIP administration relative to vehicle-treated controls (Figure 5O). Collectively, these data demonstrate that peripheral administration of acyl-GIP decreased body weight through inhibition of food intake, enhanced fatty acid oxidation, and decreased metabolizable energy, without affecting energy expenditure or BAT function. The observation that inhibition of food intake after administration of acyl-GIP correlates with increased c-FOS in the hypothalamic ARC and VMH suggests that GIP regulation of food intake is centrally regulated.

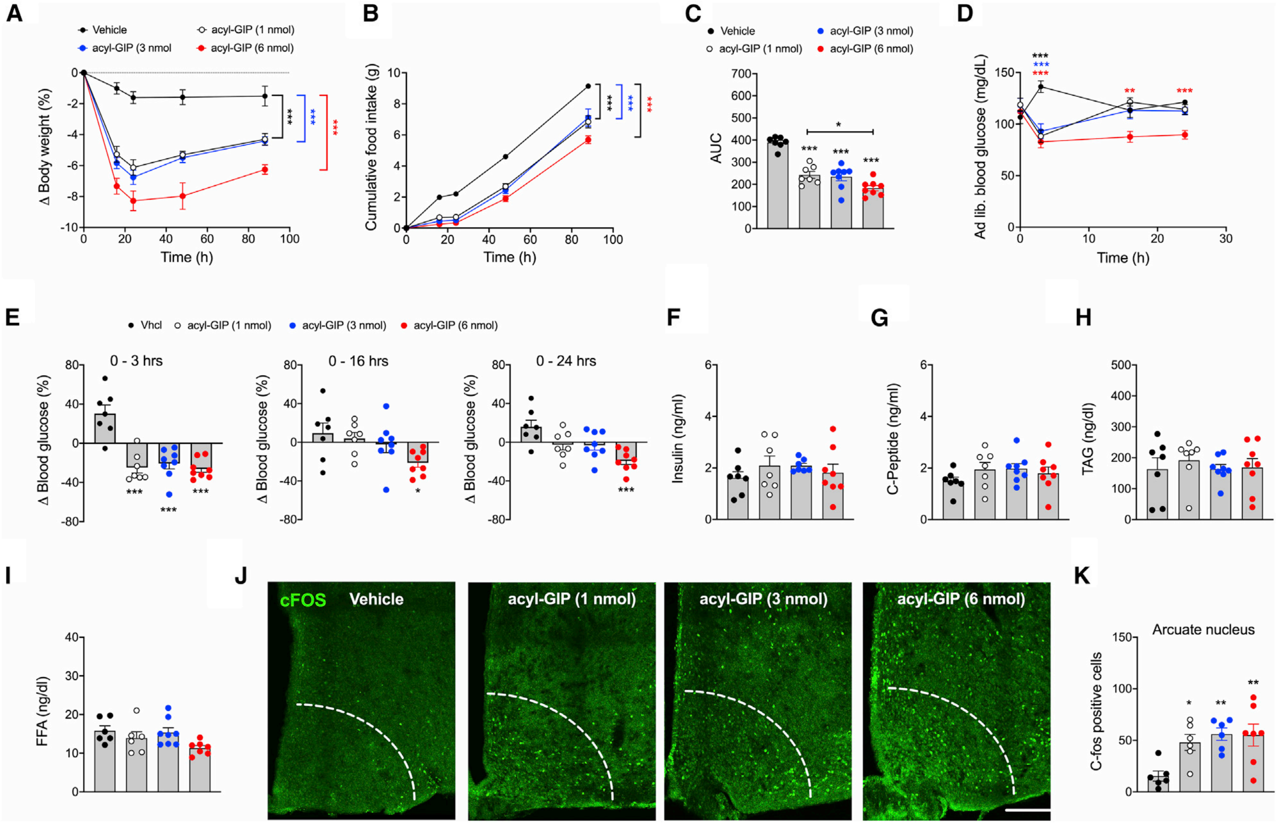

Figure 3. Acute central administration of acyl-GIP improves body weight, food intake, and glycemia in DIO mice

(A–E) Body weight change (A), food intake (B and C), and plasma levels of blood glucose (D and E) in male DIO mice treated centrally (i.c.v.) with a single dose of 1, 3, or 6 nmol acyl-GIP (N = 7–8 mice each genotype).

(F–I) *Ad libitum* plasma levels of insulin (F) and c-peptide (G) and plasma levels of triglycerides (H) and free fatty acids (I) in 32-week-old DIO mice (N = 6–8 each group).

(J and K) cFOS immunofluorescence (J) and cFOS quantification (N = 6–7 mice each genotype) (K) in the hypothalamic arcuate nucleus (ARC) of DIO mice treated with acyl-GIP.

Data represent means ± SEM. *p < 0.05, **p < 0.01, and ***p < 0.001. Scale bar, 100 μm. Longitudinal data (A, B, and D) were analyzed using two-way ANOVA with time and genotype as co-variables and Bonferroni post hoc analysis for individual time points. Bar graphs in (C), (E)–(I), and (K) were analyzed using one-way ANOVA.

Chronic peripheral administration of fatty acyl-GIP reduced body weight, food intake, and glycemia via CNS-GIPR signaling

We next assessed whether the metabolic effects of peripherally administered fatty acyl-GIP (Figure S1G) depend on CNS-GIPR signaling. While chronic daily s.c. treatment with fatty acyl-GIP (100 nmol/kg/day) decreased body weight in DIO WT mice, this effect was blunted in the CNS-*Gipr* KO mice (Figures 6A and 6B). Interestingly, while the body weight-lowering effect of peripherally administered fatty acyl-GIP is blunted, but not completely absent, in CNS-*Gipr* KO mice, we saw no effect of fatty acyl-GIP on food intake in CNS-*Gipr* KO mice (Figures 6C and 6D), implicating non-food-intake-related mechanisms independent of CNS Gipr signaling that contribute to GIPR agonism-mediated body weight lowering. These data are thus consistent with our observation that peripherally applied GIP not only decreases food intake, but also decreases metabolizable energy (Figures 5J and 5K). Importantly, fatty acyl-GIP completely loses its effects on body weight and food intake in global germline *Gipr* KO mice (Figures 6E–6H), but shows preserved effects on reduction of body weight and food intake in *GLP-1R* KO mice (Figures 6I–6L). These data suggest that the CNS-GIPR independent weight-lowering effect of fatty acyl-GIP, i.e., the decrease in metabolizable energy, may be mediated via peripheral GIPR signaling, independent of GLP-1R signaling.

We also evaluated whether CNS-GIPR signaling contributed to the metabolic benefits of a unimolecular fatty acylated GLP-1/GIP dual-agonist (Finan et al., 2013). Daily peripheral (s.c.) treatment of DIO mice with fatty acyl-GLP-1/GIP (MAR709, 10 nmol/kg/day) for 12 days decreased body weight with superior efficacy relative to a pharmacokinetically matched (IUB1746; Figure S1G) fatty acyl-GLP-1 mono-agonist (p < 0.0001; Figure 7A). The greater body weight loss in mice treated with the dual-agonist was accompanied by a greater decrease in body fat mass (Figure 7B) without difference in lean tissue mass (Figure 7C) and a greater decrease in food intake and improved glucose tolerance relative to treatment with fatty acyl-GLP-1 alone (Figures 7D–7F). While GLP-1 fully retained its ability to

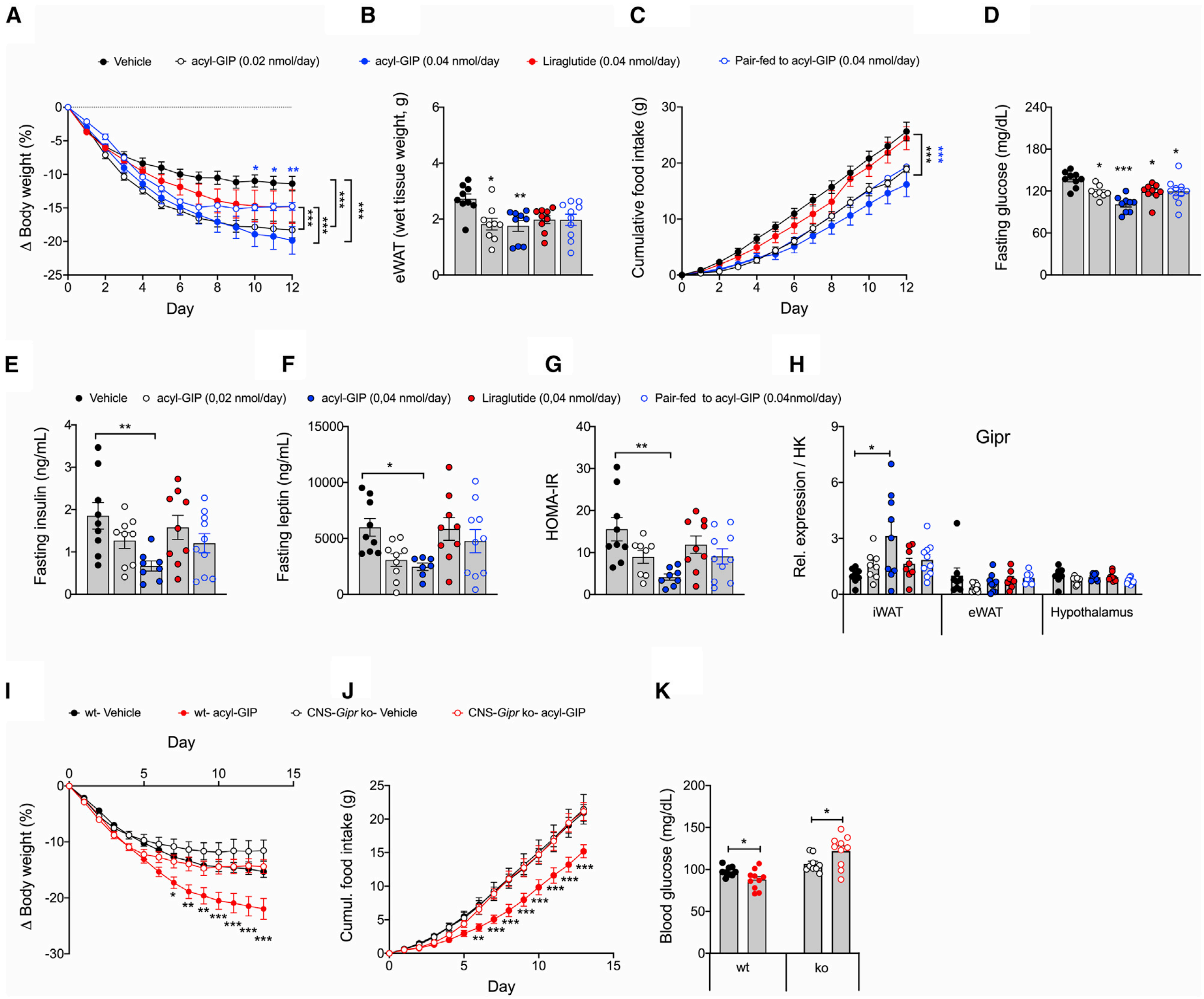

Figure 4. Chronic central administration of acyl-GIP improves body weight, food intake, and glycemia in HFD-fed WT mice, but not in CNS-*Gipr* KO mice

(A–C) Body weight (A), eWAT weight (B), and food intake (C) of HFD-fed mice treated with acyl-GIP (0.02 or 0.04 nmol/day) or liraglutide (0.04 nmol/day) or that were pair-fed to the acyl-GIP (0.04 nmol/day)-treated mice (N = 9–10 each group).

(D–G) Fasting plasma levels of blood glucose (D), insulin (E), leptin (F), and HOMA-IR (G) after 14 days of treatment (N = 7–10 mice each group).

(H) Expression of *Gipr* in iWAT, eWAT, and hypothalamus after 14 days of treatment (N = 7–10 mice each group).

(I–K) Body weight change (I), food intake (J), and fasting blood glucose (K) in HFD-fed WT and CNS-*Gipr* KO mice following treatment with 0.02 nmol/day of acyl-GIP (N = 9–10 mice each genotype).

Data represent means ± SEM. *p < 0.05, **p < 0.01, and ***p < 0.001. Longitudinal data (A, C, I, and J) were analyzed using two-way ANOVA with time and genotype as co-variables and Bonferroni post hoc analysis for individual time points. Bar graphs in (B), (D)–(H), and (K) were analyzed using one-way ANOVA.

improve body weight, fat mass, and food intake in the CNS-*Gipr* KO mice, the GLP-1/GIP dual-agonist lost its superior potency over GLP-1 (Figures 7A–7F). Of note, the GLP-1/GIP dual-agonist also equally improved glucose tolerance in WT and CNS-*Gipr* KO mice (Figures 7E and 7F), which is consistent with the shown preservation of islet *Gipr* expression (Figure 1U) and the demonstration of fully preserved insulinotropic action of GIP and GLP-1 in the islets of the CNS-*Gipr* KO mice (Figure 1W). Together, these data show that the GLP-1/GIP dual-agonist improves body weight and food intake via the CNS GIPR and improves glucose handling via peripheral mechanisms. Notably, these data further demonstrate that the CNS-*Gipr* KO mice do not show alterations in the responsiveness to GLP-1 treatment.

DISCUSSION

Here we report that body weight and glucose control are improved in HFD-fed mice with CNS deletion of either *mGipr* or *hGIPR* and that central loss of *Gipr* coincides with decreased food intake without alterations in energy expenditure. The observation that CNS-*Gipr* KO mice are protected from diet-induced obesity is consistent with the phenotype seen in global germline

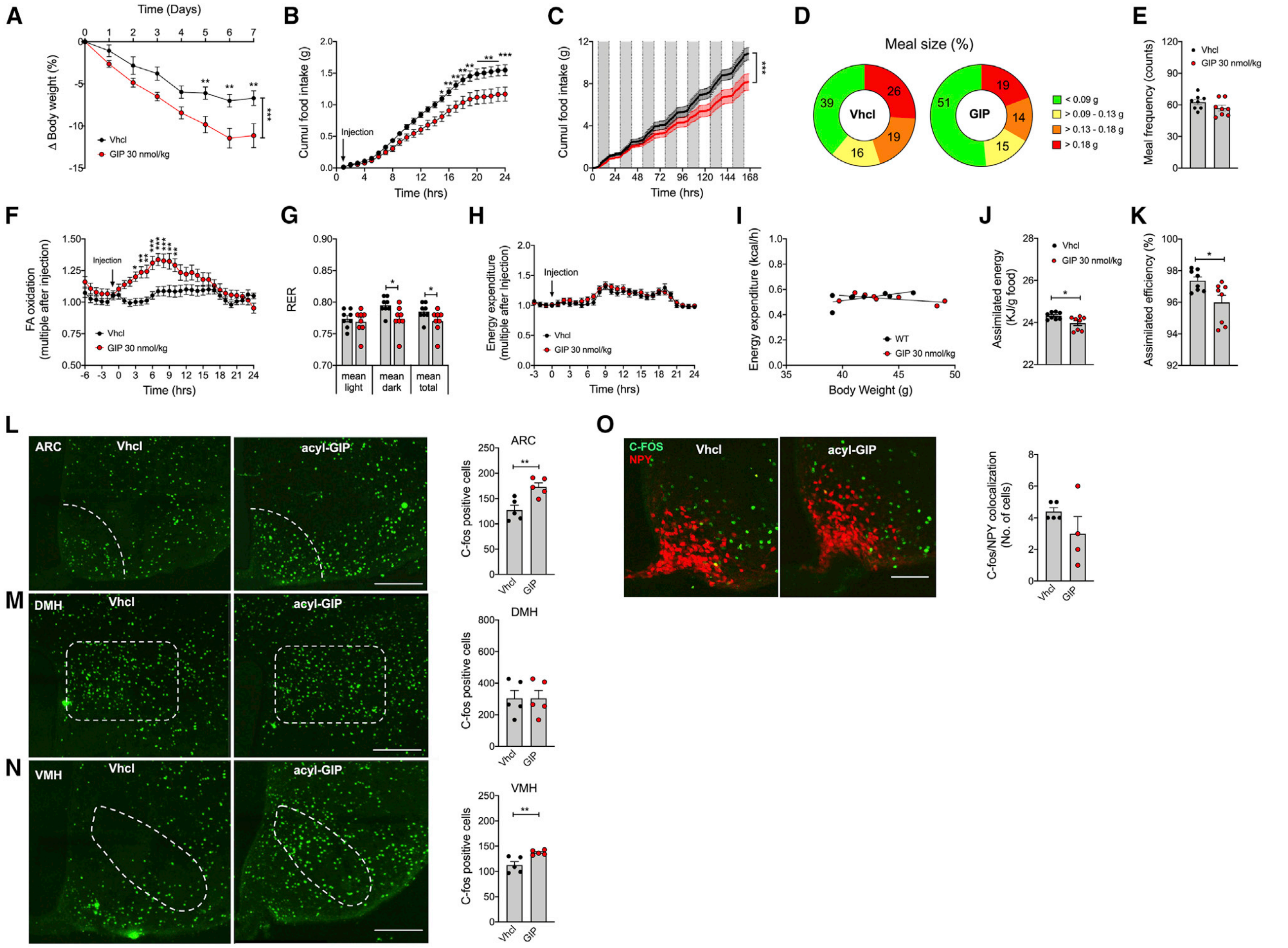

Figure 5. Peripheral administration of acyl-GIP decreases food intake and activates cFOS in the hypothalamic ARC and VMH in DIO mice

(A–C) Body weight (A) and acute (B) and chronic (C) effects of peripherally (s.c.) administered acyl-GIP (30 nmol/kg/day) on food intake in 21-week-old male DIO mice (N = 8 mice each group).

(D–I) Meal size (D) and frequency (E), acute acyl-GIP effects on fatty acid oxidation (F), respiratory exchange ratio (RER) (G), and acute and chronic effects of acyl-GIP on energy expenditure (H and I) in 21-week-old male DIO mice (N = 8 mice each group).

(J and K) Assimilated energy (J) and assimilation efficiency (K) in mice chronically treated daily s.c. for 7 days with acyl-GIP (N = 8 mice each group).

(L–O) Staining and quantification of cFOS in the ARC (L), DMH (M), and VMH (N) and cFOS/NPY co-staining (O) in the ARC of 19-week-old male HFD-fed NPY-GFP mice treated with a single peripheral (s.c.) injection of either vehicle or acyl-GIP (30 nmol/kg) (N = 5 mice each group; scale bar, 100 μm).

Data represent means ± SEM. *p < 0.05, **p < 0.01, and ***p < 0.001. Longitudinal data (A–C, F, and H) were analyzed using two-way ANOVA with time and genotype as co-variables and Bonferroni post hoc analysis for individual time points. Bar graphs in (E), (G), and (J)–(O) were analyzed using two-tailed, two-sided t test. Data in (I) was analyzed using ANCOVA with body weight as co-variate.

Gipr KO mice (Miyawaki et al., 2002) and suggests that central GIPR signaling plays a relevant role in regulating energy metabolism. It would be interesting to assess in future studies whether ablation of *Gipr* later in life leads to a metabolic phenotype comparable to what has been reported using pharmacological inhibition of GIPR. It would be interesting to assess whether the point in time when *Gipr* is ablated affects the susceptibility for body weight gain later in life. An epigenetic influence on the susceptibility to diet-induced obesity and insulin resistance has previously been shown for C57BL/6N mice (Huypens et al., 2016). Nonetheless, there remains great uncertainty as to whether GIPR activity should be activated or inhibited for the treatment of obesity and T2DM. This confusion stems from experimental evidence demonstrating that both GIPR agonists and antagonists improve body weight and glucose control in animal models of obesity. Unimolecular GLP-1/GIP dual-agonists lead, relative to GLP-1 alone, to greater improvement of body weight and glucose control in obese animals and humans (Coskun et al., 2018; Finan et al., 2013; Frias et al., 2018). In this regard, it has been hypothesized that the GIP entity of the GLP-1/GIP dual-agonists might accelerate GLP-1 receptor signaling (Holst and Rosenkilde, 2020). But notably, our data show that GLP-1/GIP loses its superior potency over GLP-1 in CNS-*Gipr* KO mice, and while GIP still lowers body weight and food intake in *GLP-1R* KO mice, it fails to affect body weight and food intake in global *Gipr* KO mice. These data collectively argue that GLP-1/GIP improves body weight and food intake via CNS GIPR signaling.

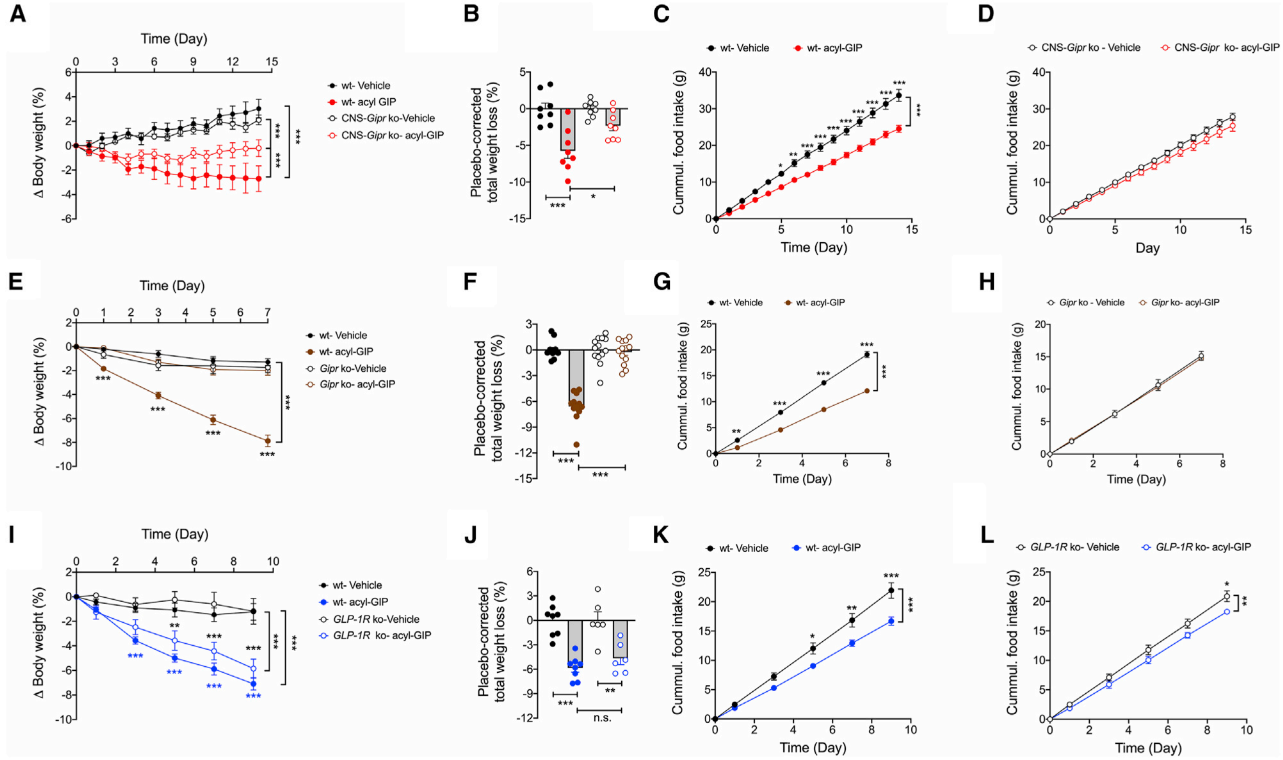

Figure 6. Chronic peripheral administration of acyl-GIP improves body weight, food intake, and glycemia via CNS-GIPR signaling

(A–D) Body weight change (A), placebo-corrected total body weight loss (B), and food intake (C and D) of HFD-fed WT and CNS-*Gipr* KO mice treated with 100 nmol/kg/day of acyl-GIP (N = 8 mice each group).

(E–H) Body weight change (E), placebo-corrected total body weight loss (F), and food intake (G and H) of HFD-fed WT and global *Gipr* KO mice treated with 100 nmol/kg/day of acyl-GIP (N = 12–13 mice each group).

(I–L) Body weight change (I), placebo-corrected total body weight loss (J), and food intake (K and L) of HFD-fed WT and global *GLP-1R* KO mice treated with 100 nmol/kg/day of acyl-GIP (N = 6–8 mice each group).

Data represent means ± SEM. *$p < 0.05$, **$p < 0.01$, and ***$p < 0.001$. Longitudinal data (A, C–E, G–I, K, and L) were analyzed using two-way ANOVA with time and genotype as co-variables and Bonferroni post hoc analysis for individual time points. Bar graphs in (B), (F), and (J) were analyzed using one-way ANOVA.

Several other hypotheses have recently been proposed to potentially underly the seemingly conflicting observation that GIPR activation also decreases body weight. It has been suggested that GIPR agonists might lower body weight through decreasing *Gipr* expression and hence through functional GIPR antagonism (Holst and Rosenkilde, 2020). Arguing against this hypothesis, we show in our manuscript that chronic central administration of acyl-GIP lowers body weight and food intake in DIO mice without changes in *Gipr* expression in the hypothalamus, eWAT, or iWAT. Furthermore, we show that single central administration of acyl-GIP is sufficient to lower body weight and food intake and to acutely induce neuronal activation (cFOS) in key hypothalamic feeding centers. These data collectively indicate that the ability of acyl-GIP to decrease body weight and food intake is mediated via CNS GIPR signaling and is unlikely driven by functional GIPR antagonism. Relevant hypotheses may further include observations that antagonizing GIPR signaling enhances the anorectic effect of leptin (Kaneko et al., 2019) and that GIPR antagonism improves WAT blood flow and nutrient supply (Asmar et al., 2017; Samms et al., 2020). Hence, it is possible that the body weight-lowering effect of GIPR antagonism resides in peripheral mechanisms on the adipose tissue while central GIPR agonism decreases body weight through centrally mediated inhibition of food intake. In line with this notion, we show that single central (i.c.v.) administration of fatty acyl-GIP improves body weight and glycemia in DIO mice and that this coincides with decreased food intake and acute neural activation (measured as cFOS) in key feeding centers of the hypothalamus, including the ARC, DMH, VMH, and LH. Similar hypothalamic cFOS patterns are also observed upon acute peripheral administration of acyl-GIP. These data hence indicate that CNS GIPR plays a direct relevant role in regulating systemic energy metabolism in mice, and pharmacologically activating this receptor decreases food intake and body weight. The data are consistent with previous findings showing presence of *Gipr* in these hypothalamic areas (Adriaenssens et al., 2019) and indicate that these acute effects of GIPR agonism are centrally mediated and unlikely driven by receptor desensitization or functional antagonism. Consistent with a relevant role of CNS GIPR signaling, chronic central administration of acyl-GIP decreases body weight and food intake, and improves glycemia in DIO WT mice with no effect of acyl-GIP on body weight, food intake, or glycemia in CNS-*Gipr* KO mice. These data thus confirm that centrally administered acyl-GIP decreases body weight and food intake via the CNS GIP receptor.

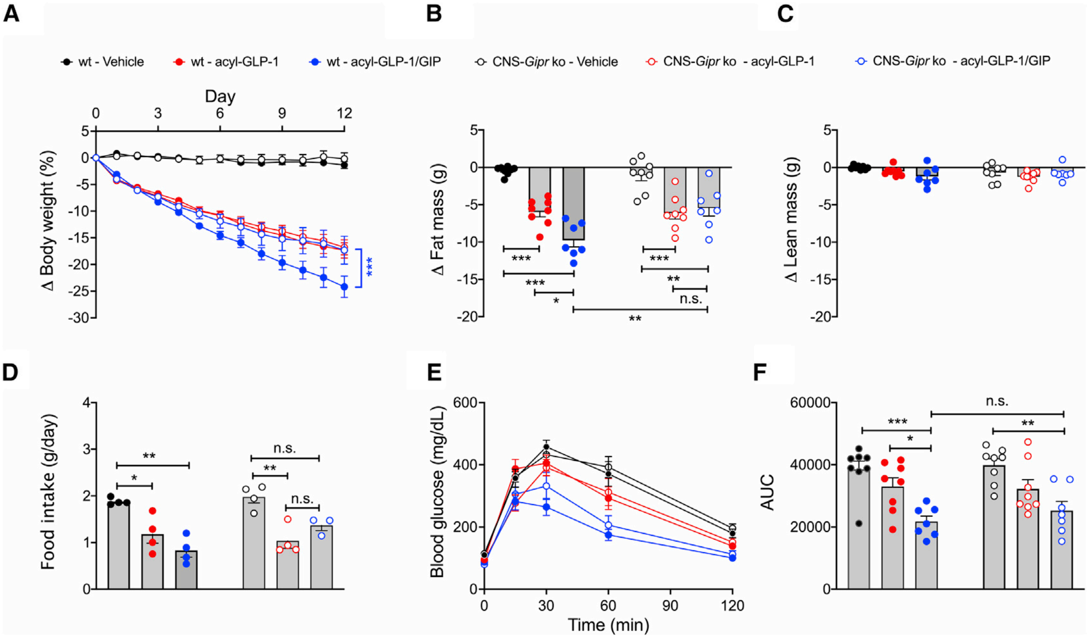

Figure 7. GLP-1/GIP loses superior potency over GLP-1 upon chronic peripheral treatment in CNS-*Gipr* KO mice

(A–D) Change in body weight (A), fat mass (B), lean mass (C), and food intake (D) of HFD-fed WT and CNS-*Gipr* KO mice treated with acyl-GLP-1 or GLP-1/GIP (MAR709) at a dose of 10 nmol/kg/day (N = 7–8 mice each group).

(E and F) Intraperitoneal glucose tolerance after 12 days of treatment (N = 7–8 mice each group).

Data represent means ± SEM. *p < 0.05, **p < 0.01, and ***p < 0.001. Longitudinal data (A and E) were analyzed using two-way ANOVA with time and genotype as co-variables and Bonferroni post hoc analysis for individual time points. Bar graphs in (B)–(D) and (F) were analyzed using one-way ANOVA.

Notably, CNS-*Gipr* KO mice are not hypersensitive to GLP-1 treatment and do not show alterations in *Gipr* expression in the pancreatic islets. Consistent with this, both incretin hormones show preserved ability to stimulate islet insulin secretion in CNS-*Gipr* KO mice. Improvement of systems metabolism by centrally administered acyl-GIP is also not related to Gipr downregulation in the hypothalamus or the adipose tissue, again suggesting that improvement of systems metabolism by acyl-GIP is not related to reduced GIPR activity. Interestingly, while centrally administered acyl-GIP fails to affect body weight and food intake in CNS-*Gipr* KO mice, peripherally administered acyl-GIP shows blunted, but not completely absent, weight lowering efficacy in CNS-*Gipr* KO mice. Consistent with this, we see both reduction of food intake and assimilation efficiency following peripheral acyl-GIP treatment in DIO mice. The latter might be associated with the ability of GIP to inhibit gastric motility. While these data indicate that acyl-GIP lowers body weight via CNS GIPR-dependent and -independent mechanisms, we see no effect of peripherally administered acyl-GIP on food intake in the CNS-*Gipr* KO mice. Thus, acyl-GIP decreases body weight via CNS-GIPR-mediated regulation of food intake and beyond this via mechanisms not related to food intake that are independent of CNS GIPR. These data are also in line with the greater decease in body weight of acyl-GIP-treated mice relative to pair-fed controls. Nonetheless, acyl-GIP shows no effect on body weight, food intake, or glycemia in global *Gipr* KO mice, but preserved effects in *GLP-1R* KO mice. The non-CNS-GIPR effects of acyl-GIP on body weight are thus mediated via peripheral GIPR signaling and unrelated to GLP-1R signaling. Potential beneficial GIP effects mediated by peripheral GIPR agonism also include the increase of lipid buffering in the WAT to protect from metabolic derangements that might result from lipid spill-over and ectopic lipid deposition in peripheral tissues (Samms et al., 2020). Of note, the GLP-1/GIP dual-agonist (MAR709) loses its superior efficacy on body weight and food intake over GLP-1 in the CNS-*Gipr* KO mice, thus indicating that this dual-agonist acts in part via the CNS-GIPR to improve systems metabolism. These data hence further argue that the dual-agonist does not improve metabolism exclusively by enhanced GLP-1R signaling. In summary, our data establish that CNS Gipr signaling is of essential relevance for systemic energy metabolism control and for the metabolic efficacy of GIP-based pharmacotherapies.

Limitations of study

We report the metabolic phenotype of mice in which GIPR has been deleted using mice that express the cre recombinase under control of the nestin promoter. While our data show selective reduction of *Gipr* expression in the hypothalamus, but not in isolated islets, bone marrow, or WAT and BAT, it is known that nestin can also to some extent be expressed external to the CNS (Harno et al., 2013). While our data clearly demonstrate that CNS GIPR signaling plays an important role in the regulation of energy metabolism, we can (like in many other cre models) not fully exclude a certain degree of off-target excision in certain cells/tissues external to the CNS. We notably see only borderline detectable expression of *Gipr* in the WAT and BAT. While this is consistent with previous studies (Adriaenssens et al., 2019), mechanistic

attribution of findings to tissues expressing low levels of receptor expression should be done with caution. Also, the lack of sufficiently sensitive and specific antibodies to quantify GIPR protein is a limitation of the study. Notably, nestin cre mice are reported in some studies to have lower lean mass and reduced body length (Harno et al., 2013). In our studies we therefore used nestin cre$^{+/-}$ *mGipr*$^{wt/wt}$ mice as controls for the nestin cre$^{+/-}$ *mGipr*$^{flx/flx}$ mice. For the studies using the nestin cre$^{+/-}$ *hGIPR*$^{flx/flx}$ mice, we confirmed that nestin cre$^{-/-}$ hGipr$^{flx/flx}$ mice do not differ in either body weight or blood glucose from nestin cre$^{+/-}$ hGipr$^{wt/wt}$ and nestin cre$^{-/-}$ hGipr$^{wt/wt}$ mice. Finally, it has to be noted that we used for our studies a fatty acid acylated GIP (Figure S1D), which despite being mechanistically comparable to native GIP (Mroz et al., 2019), potentially differs from the native peptide in both the *in vivo* potency and pharmacokinetics.

STAR★METHODS

Detailed methods are provided in the online version of this paper and include the following:

- KEY RESOURCES TABLE
- RESOURCE AVAILABILITY
  - Lead Contact
  - Materials Availability
  - Data and Code Availability
- EXPERIMENTAL MODEL AND SUBJECT DETAILS
  - Animals and housing conditions
- METHOD DETAILS
  - Indirect calorimetry
  - Bomb calorimetry
  - Preparation of RNA and gene expression analysis
  - Intracerebroventricular (icv) drug treatment
  - Drug treatment and body composition
  - Meal size and feeding patterns
  - Analysis of plasma samples
  - Islet isolation
  - Glucose-stimulated insulin secretion (sGSIS)
  - Immunohistochemistry
  - Histopathology
  - Seahorse respirometry
- QUANTIFICATION AND STATISTICAL ANALYSIS

SUPPLEMENTAL INFORMATION

Supplemental Information can be found online at https://doi.org/10.1016/j.cmet.2021.01.015.

ACKNOWLEDGMENTS

This work was supported in part by funding from the Alexander von Humboldt Foundation, the Helmholtz Alliance ICEMED and the Helmholtz Initiative on Personalized Medicine iMed by Helmholtz Association, and the Helmholtz cross-program topic Metabolic Dysfunction. This work was further supported by grants from the German Research Foundation (TRR152, TRR296, and SFB1123), the European Research Council (ERC AdG *HypoFlam* no. 695054 and ERC STG [AstroNeuroCrosstalk no. 757393]), and the German Center for Diabetes Research (DZD e.V.). D.J.D. is supported by CIHR grant 154321, a BBDC-Novo Nordisk Chair in Incretin Biology, and the Sinai Health System-Novo Nordisk Foundation Fund in Gut Hormone Physiology.

AUTHOR CONTRIBUTIONS

Q.Z. and C.T.D. co-conceptualized the project, designed and performed experiments, analyzed and interpreted data, and co-wrote the manuscript. M.B., L.S., E.M., C.L.H., G.C., G.G., A.H., M.K., K.K., B.L., M.T.M., C.S., S.K., M.J., A.B., and D.L. performed experiments and/or analyzed and interpreted data. X.L. performed experiments and participated in data analysis and interpretation. P.J.K. and S.A.M. participated in drug development and interpretation of the data. C.G.C., A.F., H.L., D.P.-T., and K.S. supervised *in vivo* studies, analyzed and interpreted data, and critically revised the manuscript. G.L., R.A., and D.J.D. provided animal models and critically revised the manuscript. S. Herzig, S. Hofmann, B.F., R.D.D., M.H.T., and C.W. co-conceptualized the project, interpreted data, and revised the manuscript. T.D.M. co-conceptualized the project, supervised the experiments, analyzed and interpreted data, and wrote the manuscript.

DECLARATION OF INTERESTS

M.H.T. is a member of the scientific advisory board of ERX Pharmaceuticals, Cambridge, MA. He was a member of the Research Cluster Advisory Panel (ReCAP) of the Novo Nordisk Foundation between 2017 and 2019. He attended a scientific advisory board meeting of the Novo Nordisk Foundation Center for Basic Metabolic Research, University of Copenhagen, in 2016. He received funding for his research projects from Novo Nordisk (2016–2020) and Sanofi-Aventis (2012–2019). He was a consultant for Bionorica SE (2013–2017), Menarini Ricerche S.p.A. (2016), and Bayer Pharma AG Berlin (2016). As former Director of the Helmholtz Diabetes Center and the Institute for Diabetes and Obesity at Helmholtz Zentrum München (2011–2018), and since 2018, as CEO of Helmholtz Zentrum München, he has been responsible for collaborations with a multitude of companies and institutions worldwide. In this capacity, he discussed potential projects with and has signed/signs contracts for his institute(s) and for the staff for research funding and/or collaborations with industry and academia worldwide, including, but not limited to, pharmaceutical corporations like Boehringer Ingelheim, Eli Lilly, Novo Nordisk, Medigene, Arbormed, BioSyngen, and others. In this role, he was/is further responsible for commercial technology transfer activities of his institute(s), including diabetes-related patent portfolios of Helmholtz Zentrum München as, e.g., WO/2016/188932 A2 or WO/2017/194499 A1. M.H.T. confirms that to the best of his knowledge none of the above funding sources were involved in the preparation of this paper. T.D.M. and K.S. receive research funding from Novo Nordisk, but these funds are unrelated to the here described work. D.J.D. has received speaking and consulting fees from Merck and Novo Nordisk Inc. and consulting fees from Forkhead Biopharmaceuticals and Kallyope Inc. R.D.D. is a co-inventor on intellectual property owned by Indiana University and licensed to Novo Nordisk. He was previously employed by Novo Nordisk. P.J.K., S.A.M., and B.F. are current employees of Novo Nordisk.

Received: July 14, 2020
Revised: December 4, 2020
Accepted: January 14, 2021
Published: February 10, 2021

REFERENCES

Adriaenssens, A.E., Biggs, E.K., Darwish, T., Tadross, J., Sukthankar, T., Girish, M., Polex-Wolf, J., Lam, B.Y., Zvetkova, I., Pan, W., et al. (2019). Glucose-dependent insulinotropic polypeptide receptor-expressing cells in the hypothalamus regulate food intake. Cell Metab. *30*, 987–996.e6.

Asmar, M., Asmar, A., Simonsen, L., Gasbjerg, L.S., Sparre-Ulrich, A.H., Rosenkilde, M.M., Hartmann, B., Dela, F., Holst, J.J., and Bülow, J. (2017). The gluco- and liporegulatory and vasodilatory effects of glucose-dependent insulinotropic polypeptide (GIP) are abolished by an antagonist of the human GIP receptor. Diabetes *66*, 2363–2371.

Bastidas-Ponce, A., Tritschler, S., Dony, L., Scheibner, K., Tarquis-Medina, M., Salinno, C., Schirge, S., Burtscher, I., Böttcher, A., Theis, F.J., et al. (2019). Comprehensive single cell mRNA profiling reveals a detailed roadmap for pancreatic endocrinogenesis. Development *146*, dev173849.

Beck, B., and Max, J.P. (1986). Direct metabolic effects of gastric inhibitory polypeptide (GIP): dissociation at physiological levels of effects on insulin-stimulated fatty acid and glucose incorporation in rat adipose tissue. Diabetologia *29*, 68.

Campbell, J.E., Ussher, J.R., Mulvihill, E.E., Kolic, J., Baggio, L.L., Cao, X., Liu, Y., Lamont, B.J., Morii, T., Streutker, C.J., et al. (2016). TCF1 links GIPR signaling to the control of beta cell function and survival. Nat. Med. *22*, 84–90.

Christensen, M., Vedtofte, L., Holst, J.J., Vilsbøll, T., and Knop, F.K. (2011). Glucose-dependent insulinotropic polypeptide: a bifunctional glucose-dependent regulator of glucagon and insulin secretion in humans. Diabetes *60*, 3103–3109.

Coskun, T., Sloop, K.W., Loghin, C., Alsina-Fernandez, J., Urva, S., Bokvist, K.B., Cui, X., Briere, D.A., Cabrera, O., Roell, W.C., et al. (2018). LY3298176, a novel dual GIP and GLP-1 receptor agonist for the treatment of type 2 diabetes mellitus: from discovery to clinical proof of concept. Mol. Metab. *18*, 3–14.

Eckel, R.H., Fujimoto, W.Y., and Brunzell, J.D. (1979). Gastric inhibitory polypeptide enhanced lipoprotein lipase activity in cultured preadipocytes. Diabetes *28*, 1141–1142.

Finan, B., Ma, T., Ottaway, N., Müller, T.D., Habegger, K.M., Heppner, K.M., Kirchner, H., Holland, J., Hembree, J., Raver, C., et al. (2013). Unimolecular dual incretins maximize metabolic benefits in rodents, monkeys, and humans. Sci. Transl. Med. *5*, 209ra151.

Finan, B., Müller, T.D., Clemmensen, C., Perez-Tilve, D., DiMarchi, R.D., and Tschöp, M.H. (2016). Reappraisal of GIP pharmacology for metabolic diseases. Trends Mol. Med. *22*, 359–376.

Frias, J.P., Nauck, M.A., Van, J., Kutner, M.E., Cui, X., Benson, C., Urva, S., Gimeno, R.E., Milicevic, Z., Robins, D., and Haupt, A. (2018). Efficacy and safety of LY3298176, a novel dual GIP and GLP-1 receptor agonist, in patients with type 2 diabetes: a randomised, placebo-controlled and active comparator-controlled phase 2 trial. Lancet *392*, 2180–2193.

Harno, E., Cottrell, E.C., and White, A. (2013). Metabolic pitfalls of CNS Cre-based technology. Cell Metab. *18*, 21–28.

Hauner, H., Glatting, G., Kaminska, D., and Pfeiffer, E.F. (1988). Effects of gastric inhibitory polypeptide on glucose and lipid metabolism of isolated rat adipocytes. Ann. Nutr. Metab. *32*, 282–288.

Holst, J.J., and Rosenkilde, M.M. (2020). GIP as a therapeutic target in diabetes and obesity: insight from incretin co-agonists. J. Clin. Endocrinol. Metab. *105*, e2710–e2716.

Huypens, P., Sass, S., Wu, M., Dyckhoff, D., Tschöp, M., Theis, F., Marschall, S., Hrabě de Angelis, M., and Beckers, J. (2016). Epigenetic germline inheritance of diet-induced obesity and insulin resistance. Nat. Genet. *48*, 497–499.

Kaneko, K., Fu, Y., Lin, H.Y., Cordonier, E.L., Mo, Q., Gao, Y., Yao, T., Naylor, J., Howard, V., Saito, K., et al. (2019). Gut-derived GIP activates central Rap1 to impair neural leptin sensitivity during overnutrition. J. Clin. Invest. *129*, 3786–3791.

Killion, E.A., Wang, J., Yie, J., Shi, S.D., Bates, D., Min, X., Komorowski, R., Hager, T., Deng, L., Atangan, L., et al. (2018). Anti-obesity effects of GIPR antagonists alone and in combination with GLP-1R agonists in preclinical models. Sci. Transl. Med. *10*, eaat3392.

Kim, S.J., Nian, C., and McIntosh, C.H. (2007). Activation of lipoprotein lipase by glucose-dependent insulinotropic polypeptide in adipocytes. A role for a protein kinase B, LKB1, and AMP-activated protein kinase cascade. J. Biol. Chem. *282*, 8557–8567.

Kim, S.J., Nian, C., and McIntosh, C.H. (2010). GIP increases human adipocyte LPL expression through CREB and TORC2-mediated trans-activation of the LPL gene. J. Lipid Res. *51*, 3145–3157.

Kim, S.J., Nian, C., Karunakaran, S., Clee, S.M., Isales, C.M., and McIntosh, C.H. (2012). GIP-overexpressing mice demonstrate reduced diet-induced obesity and steatosis, and improved glucose homeostasis. PLoS One *7*, e40156.

Miyawaki, K., Yamada, Y., Ban, N., Ihara, Y., Tsukiyama, K., Zhou, H., Fujimoto, S., Oku, A., Tsuda, K., Toyokuni, S., et al. (2002). Inhibition of gastric inhibitory polypeptide signaling prevents obesity. Nat. Med. *8*, 738–742.

Mroz, P.A., Finan, B., Gelfanov, V., Yang, B., Tschöp, M.H., DiMarchi, R.D., and Perez-Tilve, D. (2019). Optimized GIP analogs promote body weight lowering in mice through GIPR agonism not antagonism. Mol. Metab. *20*, 51–62.

Nauck, M.A., Heimesaat, M.M., Orskov, C., Holst, J.J., Ebert, R., and Creutzfeldt, W. (1993). Preserved incretin activity of glucagon-like peptide 1 [7-36 amide] but not of synthetic human gastric inhibitory polypeptide in patients with type-2 diabetes mellitus. J. Clin. Invest. *91*, 301–307.

Pinto, S., Roseberry, A.G., Liu, H., Diano, S., Shanabrough, M., Cai, X., Friedman, J.M., and Horvath, T.L. (2004). Rapid rewiring of arcuate nucleus feeding circuits by leptin. Science *304*, 110–115.

Renner, S., Fehlings, C., Herbach, N., Hofmann, A., von Waldthausen, D.C., Kessler, B., Ulrichs, K., Chodnevskaja, I., Moskalenko, V., Amselgruber, W., et al. (2010). Glucose intolerance and reduced proliferation of pancreatic beta-cells in transgenic pigs with impaired glucose-dependent insulinotropic polypeptide function. Diabetes *59*, 1228–1238.

Samms, R.J., Coghlan, M.P., and Sloop, K.W. (2020). How may GIP enhance the therapeutic efficacy of GLP-1? Trends Endocrinol. Metab. *31*, 410–421.

Speakman, J.R., Fletcher, Q., and Vaanholt, L. (2013). The '39 steps': an algorithm for performing statistical analysis of data on energy intake and expenditure. Dis. Model. Mech. *6*, 293–301.

Tschöp, M.H., Speakman, J.R., Arch, J.R., Auwerx, J., Brüning, J.C., Chan, L., Eckel, R.H., Farese, R.V., Jr., Galgani, J.E., Hambly, C., et al. (2011). A guide to analysis of mouse energy metabolism. Nat. Methods *9*, 57–63.

Ussher, J.R., Campbell, J.E., Mulvihill, E.E., Baggio, L.L., Bates, H.E., McLean, B.A., Gopal, K., Capozzi, M., Yusta, B., Cao, X., et al. (2018). Inactivation of the glucose-dependent insulinotropic polypeptide receptor improves outcomes following experimental myocardial infarction. Cell Metab. *27*, 450–460.e6.

STAR★METHODS

KEY RESOURCES TABLE

REAGENT or RESOURCE	SOURCE	IDENTIFIER
Antibodies		
Rabbit polyclonal anti-cFos	Synaptic System	Cat# 226003;RRID: AB_2231974
Alexa Fluor 568 donkey-anti-rabbit	Thermo Fisher Scientific	Cat# A10042;RRID: AB_2534017
Chemicals, Peptides, and Recombinant Proteins		
acyl-GIP	Mroz et al., 2019	IUB0271
acyl-GLP-1	Mroz et al., 2019	IUB1746
GLP-1/GIP	Mroz et al., 2019	MAR709
Liraglutide	Finan et al., 2013	Novo Nordisk
Hematoxylin and Eosin (H&E)	Sigma-Aldrich	Cat# 517-28-2
Metamizol	HMGU Internal Pharmacy	N/A
Ketamine	HMGU Internal Pharmacy	N/A
Xylazine	HMGU Internal Pharmacy	N/A
Meloxicam	HMGU Internal Pharmacy	N/A
collagenase P	Sigma-Aldrich	Cat# 11213865001
Hanks Balanced Salt Solution (HBSS)	Thermo Fisher Scientific	Cat# 14065056
RPMI medium 1640	Sigma	Cat# R8758
Paraformaldehyde (PFA)	Sigma	Cat# 158127
Dexamethasone	Sigma	Cat# D1756
IBMX	Sigma	Cat# 15879
Indomethacine	Sigma	Cat# I7378
Rosiglitazone	VWR	Cat# CAYM71740-10
T3	Sigma	Cat# T6397
Insulin	Sigma	Cat# I9278
Oligomycin	Sigma	Cat# O4876
Dinitrophenol	Sigma	Cat# 34334
Rotenone	Sigma	Cat# R8875
Isoproterenol	Sigma	Cat# I6504
Antimycin A	Sigma	Cat# A8674
Critical Commercial Assays		
RNase Kit	QIAGEN	Cat# 74106
QuantiTect reverse transcription kit	QIAGEN	Cat# 205313
SYBR Green qPCR Master Mixes	Thermo Fisher Scientific	Cat# 4364344
Ultrasensitive mouse insulin ELISA kit	Crystal Chem	Cat# 90080
Mouse C-Peptide ELISA kit	Crystal Chem	Cat# 90050
Mouse total GLP-1 ELISA kit	Crystal Chem	Cat# 81508
Mouse leptin ELISA kit	Crystal Chem	Cat# 90030
Triglyceride assay kit	Cobas Roche/Hitachi	Cat# 11489232
Triglyceride assay kit	Wako Chemical	Cat# 290-63701
Total cholesterol assay kit	Cobas Roche/Hitachi	Cat# 11877771
Total cholesterol assay kit	Wako Chemical	Cat# 993-02501
NEFA-HR(2) Assay	Wako Chemical	Cat# 91797&91898
Pierce BCA Protein assay Kit	Thermo Fisher Scientific	Cat# 23225
HbA1c kit	Axonlab	Cat#10698915
96-well Genomic DNA Kit	Favorgen	Cat# FADWE 96004
Alzet brain infusion kit 3	Alzet	Cat# 0008851

(*Continued on next page*)

Continued		
REAGENT or RESOURCE	SOURCE	IDENTIFIER
Alzet osmotic minipump (Alzet model 1002	Alzet	Cat# 0004317
Experimental Models: Organisms/Strains		
BAT primary cells harvested from naive C57BL6/J mice	This paper	N/A
Experimental Models: Cell Lines		
B6.Cg-Tg(Nes-cre)1Kln/J	The Jackson Laboratory	Cat#003771
C57BL/6J(Gipr$^{flx/flx}$)	Campbell et al., 2016	N/A
C57BL/6N(GIPR$^{flx/flx}$)	This paper	Boehringer Ingelheim
Oligonucleotides		
Primers for RT PCR, see Table S1	This paper	N/A
Software and Algorithms		
GraphPad Prism 8.0	GraphPad	N/A
GraphPad Prism 9.0	GraphPad	N/A
ImageJ	NIH Image	N/A
SPPS v. 26	IBM	N/A
Other		
TSE Phenomaster	TSE Systems	N/A

RESOURCE AVAILABILITY

Lead Contact

Further information and requests for resources and reagents should be directed to and will be fulfilled by the Lead Contact, Timo D. Müller (timo.mueller@helmholtz-muenchen.de).

Materials Availability

This study did not generate new unique reagents. The drugs used in this study were kindly provided by Novo Nordisk Research Center Indianapolis. Nestin cre mice are available from the Jackson Laboratories (Stock No. 003771).

Data and Code Availability

The study did not generate/analyze datasets/codes. The raw data underlying the figure panels are available from the Lead Contact upon reasonable request.

EXPERIMENTAL MODEL AND SUBJECT DETAILS

Animals and housing conditions

Animal experiments were performed in accordance with the Animal Protection Law of the European Union, Switzerland or the United States of America and upon permission by the state of Bavaria, Germany, the cantonal Veterinary Office Zurich, Switzerland, or the University of Cincinnati, OH, USA.

Only naive male mice were used in the studies since female mice are largely protected from diet-induced obesity and glucose intolerance.

CNS-*hGIPR* KO mice were provided by Boehringer Ingelheim and kept on a C57/BL6N background. CNS-*hGIPR* KO mice were generated by replacing mouse *Gipr* (*mGipr*) from exon 3-14 with the human *hGIPR* sequence (Taconic Biosciences GmbH, Cologne, Germany). CNS-specific *hGIPR* KO mice were generated by crossing *hGIPR*$^{flx/flx}$ mice with mice expressing the cre recombinase under control of the nestin promoter. *Nes-cre*$^{+/-}$ *hGIPR*$^{flx/flx}$ (CNS-*hGIPR* KO) and littermate *Nes-cre*$^{-/-}$ *hGIPR*$^{flx/flx}$ (WT) were considered for the experiments after confirmation that *Nes-cre*$^{-/-}$ *hGIPR*$^{flx/flx}$ mice do not differ in body weight or fasting blood glucose from *Nes-cre*$^{+/-}$ *hGIPR*$^{wt/wt}$ mice (Figures S3C and S3D). Only naive male mice were considered for the experiments. Mice were double-housed or single housed when aggressive behavior required separation. For metabolic phenotyping, naive age-matched male mice were grouped based on their genotypes. Intraperitoneal glucose tolerance test (ipGTT) was assessed in 18-week-old *CNS-hGIPR* KO mice after 6 h fasting with stimulation of 2 g glucose per kg body weight. Body composition (fat and lean mass) was assessed in 19-week-old *CNS-hGIPR* KO mice by Aloka LaTheta computed tomography (CT) scanner using LaTheta software (Zinsser Analytic, UK). The ages and sample sizes corresponding to the individual measures are indicated in the figure legends.

Nestin-cre mice were purchased from Jackson laboratories (Bar Harbor, ME, USA, cat. no. 003771) and backcrossed to C57BL/6J for > 10 generations. CNS-*mGipr* KO mice were bred on a C57BL/6J background and were generated by crossing *Gipr*$^{flx/flx}$ mice (Campbell et al., 2016) with mice expressing the Cre recombinase under control of the nestin promoter. Gipr$^{flx/flx}$ mice were cross-bred for > 5 generations to C57BL/6J. Only naive male *Nes-cre*$^{+/-}$ *Gipr*$^{flx/flx}$ (CNS-*mGipr* KO) and *Nes-cre*$^{+/-}$ *Gipr*$^{wt/wt}$ mice (WT) were considered for the study. Mice were double-housed or single housed if aggressive behavior required separation. For metabolic phenotyping (Figure 1), age-matched male mice were grouped based on their genotypes. ipGTT was assessed in 42-week-old CNS-*mGipr* KO mice after 6 h fasting with stimulation of 1.75 g glucose per kg body weight. Body composition was analyzed using a magnetic resonance whole-body composition analyzer (EchoMRI, Houston, TX). The ages and sample sizes corresponding to the individual measures are indicated in the figure legends.

For studies using regular naive diet-induced obese (DIO) mice, C57BL/6J mice were purchased from Janvier Labs (Le Genest-Saint-Isle, France). Mice were randomly assigned into treatment groups matched for body weight and body composition (fat and lean tissue mass). The ages and sample sizes corresponding to the individual measures are indicated in the figure legends.

Mice were kept at a constant environment with ambient temperature set to 22 ± 2°C with constant humidity (45 – 65%) and a 12 h/12 h light/dark cycle. For studies in *mGipr* KO mice and regular DIO mice, animals had free access to water and were fed *ad libitum* with either a standard chow (Type 1314, Altromin GmbH, Lage, Germany) or HFD (58% kcal fat; Research Diets, New Brunswick, NJ, USA; cat. no. D12331). For studies using *hGIPR* KO mice, animals had free access to water and were fed with either a standard chow diet (cat. no. 2222; Kliba-Nafag, Kaiseraugst, Switzerland) or a HFD (cat. no. 2127; Kliba-Nafag, Kliba-Nafag, Kaiseraugst, Switzerland). During the experiments, the health status of the animals was checked and scored daily and included assessment of overall behavior, skin/fur irritations, wounds and injuries, scratching behavior, piloerection or other signs of abnormal appearance.

For analysis of mitochondrial bioenergetics using seahorse technology, murine brown preadipocytes (immortalized by SV40 large T-antigen) were harvested from 8-12 week-old male chow-fed C57BL/6J mice purchased from the Jackson Laboratory (Cat. no. 000664).

METHOD DETAILS

For animal studies, sample sizes were calculated based on a power analysis assuming that a ≥ 5 g difference in body weight between genotypes can be assessed with a power of ≥ 75% when using a 2-sided statistical test under the assumption of a standard deviation of 3.5 and an alpha level of 0.05. Investigators were not blinded to genotypes and treatment conditions since all investigators need to be able at any time to show federal animal protocol approval, study designs, results, treatments as well as number and genotypes of used animals to federal authorities upon spontaneous inspections by the governmental authorities. No data were excluded from the studies unless a significant outlier was detected using a statistical outlier test (Grubbs Test; GraphPad Prizm). No animals were excluded from the studies unless health issues demanded exclusion of single mice (e.g., due to fighting injuries, dermatitis or due to detached brain cannulas).

Indirect calorimetry

Energy expenditure, substrate utilization (respiratory exchange ratio, RER) and home-cage activity were assessed in temporally single-house mice using a climate-controlled indirect calorimetric system (TSE System, Bad Homburg, Germany). After acclimatization for 24 h, levels of O_2 and CO_2 were measured every 10 min for 4 - 5 days. Indirect calorimetry was performed in HFD-fed 20-week-old CNS-*hGIPR* KO mice and 29-week-old CNS-*mGipr* KO mice. Data for energy expenditure were analyzed using ANCOVA with body weight as covariate as previously suggested (Speakman et al., 2013; Tschöp et al., 2011). Fatty acid oxidation (kcal/h) was assessed by the formula "*energy expenditure (kcal/h) x (1-RER)/0.3*."

Bomb calorimetry

Assimilated energy and assimilation efficiency was assessed using the C200 Oxygen Bomb Calorimeter (IKA, Staufen, Germany). Food consumption and feces production were measured/collected over 7 days during continuous daily peripheral (s.c.) acyl-GIP treatment. Feces and food were dried for several days at 65 °C before measuring food/fecal energy content for assessment of assimilated energy (KJ/g food).

Preparation of RNA and gene expression analysis

Total RNA was prepared using RNeasy Kit (QIAGEN, Hilden, Germany) according to manufacturer's instructions. cDNA synthesis was performed using QuantiTect Reverse Transcription Kit (QIAGEN, Hilden, Germany) according to manufacturer's instructions. Gene expression was profiled using quantitative PCR-based (qPCR) techniques using SYBR green or TaqMan Single Probes (Thermo Fisher Scientific, Erlangen, Germany). The relative expression of the selected genes was measured using the 7900HT Fast Real-Time PCR System (Thermo Fisher Scientific, Erlangen, Germany). The relative expression levels of each gene were normalized to the housekeeping gene peptidylprolyl isomerase B (*Ppib*), Hypoxanthine Phosphoribosyltransferase (*Hprt*), or Acidic ribosomal phosphoprotein P0 (*36B4*). For primer sequences see Table S1.

Intracerebroventricular (icv) drug treatment

Mice received one oral drop of Metamizol (appx. 50 μl) and were subsequently anaesthetized using ketamine (7 mg/kg) /xylazine (100 mg/kg). Mice were then treated with Lidocaine (6 mg/kg) followed by implantation of a cannula (Alzet brain infusion kit 3,

Cupertino, CA) into the lateral ventricle (anteroposterior:-0.2 mm from bregma, lateral: ± 1.0 mm to bregma and dorsoventral: 3 mm below skull) using a stereotaxic apparatus (David Kopf Instruments, USA). For the acute study, 1 μl of 0.9% saline and acyl-GIP were applied to their respective experimental group. Body weight, food intake, and ad lib blood glucose were recorded as described. For chronic drug treatment, the cannula (Alzet brain infusion kit 3, Cupertino, CA) was connected to an Alzet osmotic minipump (Alzet model 1002, Cupertino, CA; flow rate 0.25 μl/h, delivery rate 14 days) via a 2 cm-long vinyl tubing. The minipumps were filled with 0.9% saline, acyl-GIP, or liraglutide, then primed overnight at 37°C in 0.9% saline before subcutaneous implantation. After surgery, mice received meloxicam (1 mg/kg) subcutaneously every 12 h for the first post-operative days.

Drug treatment and body composition

Mice were treated daily via subcutaneous injection in a volume of 5 μl per gram body weight. Body composition was analyzed using a magnetic resonance whole-body composition analyzer (EchoMRI, Houston, TX). For glucose tolerance, levels of blood glucose were sampled from 6 h fasted mice following intraperitoneal administration of 1.75 g glucose per kilogram body weight.

Meal size and feeding patterns

Meal patterns were analyzed from food intake data collected in the calorimetric chambers using a moving average of 40 min food intake per animal. Time coherent time windows with a moving average above 0.009 g were defined as a meal. Total meal sizes were binarized into four categories: small (< 0.09), medium (> 0.09 - 0.13), large (> 0.13 - 0.18), very large (> 0.18). Significance between meal sizes were calculated using Student's t test. Single individual data points indicating shredding of food ($\geq$ 0.25 g / 10 min) were excluded from the analysis.

Analysis of plasma samples

Blood samples were collected and immediately kept on ice, centrifuged at 3000 *g* and 4°C. Plasma was stored at $-$80°C. Plasma total immunoreactive insulin, C peptide, total GLP-1 and leptin were measured using commercially available ELISA kits from Crystal Chem, Zaandam, Netherlands (Insulin cat. no. 90080; c-peptide cat. no. 90050, GLP-1 cat. no. 81508, Leptin cat. no. 90030). Plasma triglycerides were determined using commercially available kits from either Roche Diagnostics International, Switzerland (cat. no. 11489232) or Wako Chemical (Wako Pure Chemical Industries, Japan). Total cholesterol was determined using kits from either Roche Diagnostics International, Switzerland (cat. no. 11877771) or Wako Pure Chemical Industries, Japan (cat no. 993-02501). Plasma FFA levels were determined using kits from Wako Pure Chemical Industries, Japan (cat. no. 9196). All ELISAs were performed according to the manufacturer's instruction.

Islet isolation

For islet isolation, the pancreas was perfused with 6 mg/mL of collagenase P (Sigma-Aldrich, Germany, cat. no. 11213865001) and dissolved in Hanks Balanced Salt Solution (HBSS; Thermofisher Scientific, Planegg, Germany, cat. no. 14065056) with Ca^{2+}/Mg^{2+}. After applying to a gradient solution, islets were isolated and were handpicked under the microscope. Islets were kept overnight at 37°C 5% CO_2 in culture with 11 mM glucose in RPMI medium 1640 (Sigma Aldrich, Taufkirchen, Germany, cat no. R8758) supplemented with 10% (vol/vol) FBS Heat Inactivated, 1% (vol/vol) penicillin and streptomycin (Bastidas-Ponce et al., 2019).

Glucose-stimulated insulin secretion (sGSIS)

The islets were transferred to a 96-well plate, and the culture media was replaced with Krebs-Ringer bicarbonate buffer (Sigma Aldrich, Taufkirchen, Germany, cat. no. K4002) modified with HEPES (KRBH) containing 129 mM NaCl, 4.8 mM KCl, 1.2 mM KH2PO4, 1.2 mM MgSO4·H2O, 2 mM CaCl2, 24 mMNaHCO3, 6 mM HEPES, and 0.2% bovine serum albumin with PH adjusted to 7.4. Then, the islets were incubated for 1 h in starving glucose media (KRBH with 1 mM Glucose) before starting the GSIS. Different glucose concentrations (2.8 and 16.8 mM) were added to the islets (for 1 h each). For compound treatment, a concentration of 10 nM of GLP-1 or GIP was added during the GSIS. The supernatants were collected and used for insulin measurement. Islets were lysed for DNA measurements. Insulin levels were measured with the ultrasensitive insulin ELISA kit (Crystal Chem, Zaandam, Netherlands, cat. no. 90080). The data was normalized to the DNA content.

Immunohistochemistry

For cFOS staining, HFD-fed WT or NPY-GFP mice (Pinto et al., 2004) were anesthetized with CO_2 1.5 h after either central (icv) or peripheral (s.c.) injection of the acyl-GIP, and transcardially perfused with phosphate-buffered saline (PBS) followed by 4% neutral buffered paraformaldehyde (PFA). Brains were harvested and equilibrated in 30% sucrose for three days, and sliced on a cryostat in the coronal plane at 30 μm. Slices were washed 5 times with 0.5% Triton X-100 in tris-buffered saline (TBS) followed by 1 h block with SUMI (0.25% gelatin and 0.5% Triton X-100 in TBS). After blocking, slices were incubated overnight with primary antibody anti-cFOS (Synaptic system, Goettingen, Germany; rabbit polyclonal antibody cat. no. 226003, dilution: 1:2000,) in SUMI at 4°C. After 5 times wash in TBS, slices were incubated 1 h with Alexa Fluor 568 donkey-anti-rabbit (Molecular Probes, Life Technologies GmbH, Darmstadt, Germany, dilution 1:1000) secondary antibody. After several washes, slices were mounted on gelatin-pre-coated glass slides, and coverslipped for image quantification. ImageJ was applied for quantifying cFOS postivie cells and cFOS-NPY co-localized cells. Images of single focal planes were captured at 20X magnification by a Leica SP5 scanning confocal microscope. The number of

cFOS positive nuclei within the hypothalamic area was determined according to the Allen mouse brain atlas and the analyses were performed without previous knowledge of the experimental group.

Histopathology

Mice were sacrificed by cervical dislocation. Liver and iWAT were embedded in paraffin using a vacuum infiltration processor TissueTEK VIP (Sakura), and processed as 3 or 4 μm slides using a HMS35 rotatory microtome (Zeiss) before H&E staining. For H&E staining, rehydration was done in a decreasing ethanol series, rinsing with tap water, 2 min Mayers acid Hemalum, bluing in tap water followed by 1 min EosinY (both Sigma-Aldrich, MO, USA). Dehydration was performed in an increasing ethanol series, mounting with Pertex (Medite GmbH, Burgdorf, Germany), and coverslips (Carl Roth Chemicals, Karlsruhe, Germany). The slides were evaluated independently using a brightfield microscope (Axioplan; Zeiss, Jena, Germany). Steatosis was graded by the presence of fat vacuoles in liver cells according to the percentage of affected tissue (0: < 5%; 1: 5%–33%; 2: 33%–66%; 3: > 66%); the number of samples falling into each grade divided by total sample number was considered the percentage of steatosis grade.

Seahorse respirometry

Murine brown preadipocytes (immortalized by SV40 large T-antigen) were harvested from 8-12 week old male chow-fed C57BL/6J mice, plated onto XF96 V3 PET cell culture microplates (Agilent Technologies; 12K per well) and individually differentiated. At confluency, differentiation was started using a brown fat differentiation cocktail (growth media, 5 μM dexamethasone, 0.5 mM IBMX, 125 μM indomethacine, 1 μM rosiglitazone, 5 μg/mL insulin, 1 nM T3), followed at day 2 of differentiation by exposure to continuation medium (growth media, 1 μM rosiglitazone, 5 μg/mL insulin, 1 nM T3), followed by differentiation medium (growth media, 1 μM rosiglitazone, 5 μg/mL insulin, 1 nM T3) from day 4 to 6. Before measurements of cellular respiration at day 6, the cells were washed twice with assay medium (XF DMEM + 20 mM glucose, 2 mM glutamine, 2% BSA) and then incubated in 180 μL of assay medium for 10 min without CO2 at 37°C before transfer to the XF96 Extracellular Flux analyzer (Agilent Technologies). Assay cycles were set to 2 min mixing and 2 min measuring intervals. Oligomycin (5 μg/mL) served to inhibit ATP synthase, dinitrophenol (DNP; 150 μM) to fully uncouple respiration, and a final cocktail served to correct for non-mitochondrial OCR, consisting of rotenone (5 μM) and antimycin A (2 μM) to inhibit respiratory complexes I and III, respectively, with 2-deoxyglucose (2-DG; 100 mM).to inhibit glycolytic flux.

QUANTIFICATION AND STATISTICAL ANALYSIS

All data were analyzed using the Kolmogorov-Smirnov test for normal distribution (https://www.socscistatistics.com/tests/kolmogorov/default.aspx). All data were normally distributed and met the assumption of the used statistical approaches. Statistical analyses were performed using the statistical tools implemented in GraphPad Prism8 (version 8.3.0). Differences between groups were assessed by Student's 2-sided 2-tailed t test, 1-way ANOVA or 2-way ANOVA with time and genotype as co-variants followed by Bonferroni's post hoc multiple comparison testing for individual time points. The statistical tests and sample sizes underlying the respective measures are indicated in the figure legends. All data represent means ± SEM. Asterisks indicate *$p < 0.05$, **$p < 0.01$ and ***$p < 0.001$. $p < 0.05$ was considered statistically significant. Differences in energy expenditure were calculated using ANCOVA with body weight as co-variate using SPSS (version 24).

Cell Metabolism, Volume *33*

Supplemental information

The glucose-dependent insulinotropic polypeptide (GIP) regulates body weight and food intake via CNS-GIPR signaling

Qian Zhang, Challa Tenagne Delessa, Robert Augustin, Mostafa Bakhti, Gustav Colldén, Daniel J. Drucker, Annette Feuchtinger, Cristina Garcia Caceres, Gerald Grandl, Alexandra Harger, Stephan Herzig, Susanna Hofmann, Cassie Lynn Holleman, Martin Jastroch, Susanne Keipert, Maximilian Kleinert, Patrick J. Knerr, Konxhe Kulaj, Beata Legutko, Heiko Lickert, Xue Liu, Gerd Luippold, Dominik Lutter, Emilija Malogajski, Marta Tarquis Medina, Stephanie A. Mowery, Andreas Blutke, Diego Perez-Tilve, Ciro Salinno, Laura Sehrer, Richard D. DiMarchi, Matthias H. Tschöp, Kerstin Stemmer, Brian Finan, Christian Wolfrum, and Timo D. Müller

Suppl. Figure 1 – Expression of m*Gipr* in selected tissues (Related to Figure 1) and sequence of the designated peptides (Related to Figures 3-7).

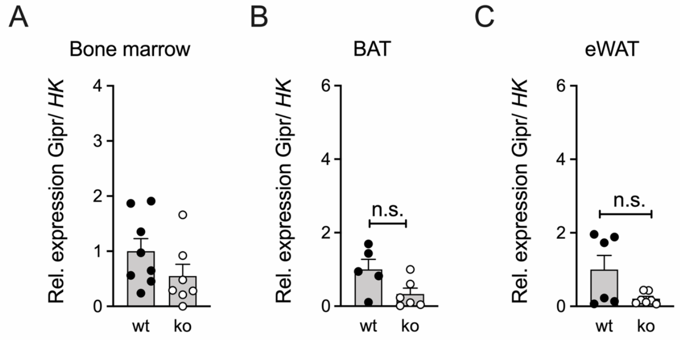

D

```
IUB 0271 (acyl-GIP)      YXEGT FISDY SIAMD KIHQQ DFVNW LLAQK GKKND WKHNI TQK (γE-γE-γE-C16)-OH
IUB 1746 (acyl-GLP-1)    HXEGT FTSDV SIYLD KQAAX EFVNW LLAGG PSSGA PPPSK(C16)
MAR709   (GLP-1/GIP)     YXEGT FTSDY SIYLD KQAAX EFVNW LLAGG PSSGA PPPSK(C16)
```

Suppl. Figure 1. Expression of m*Gipr* in selected tissues and sequence of the designated peptides. Expression of *mGipr* in bone marrow of male HFD-fed wt and CNS-*Gipr* ko mice (N=7-8 mice each group) **(a)** and in BAT **(b)** and eWAT **(c)** of male HFD-fed wt and CNS *mGipr* ko mice (N=5-7 mice each group). Peptide sequence of used molecules **(d)**.

Suppl. Figure 2 – Metabolic phenotype of Chow-fed CNS-*Gipr* ko mice (Related to Figure 1)

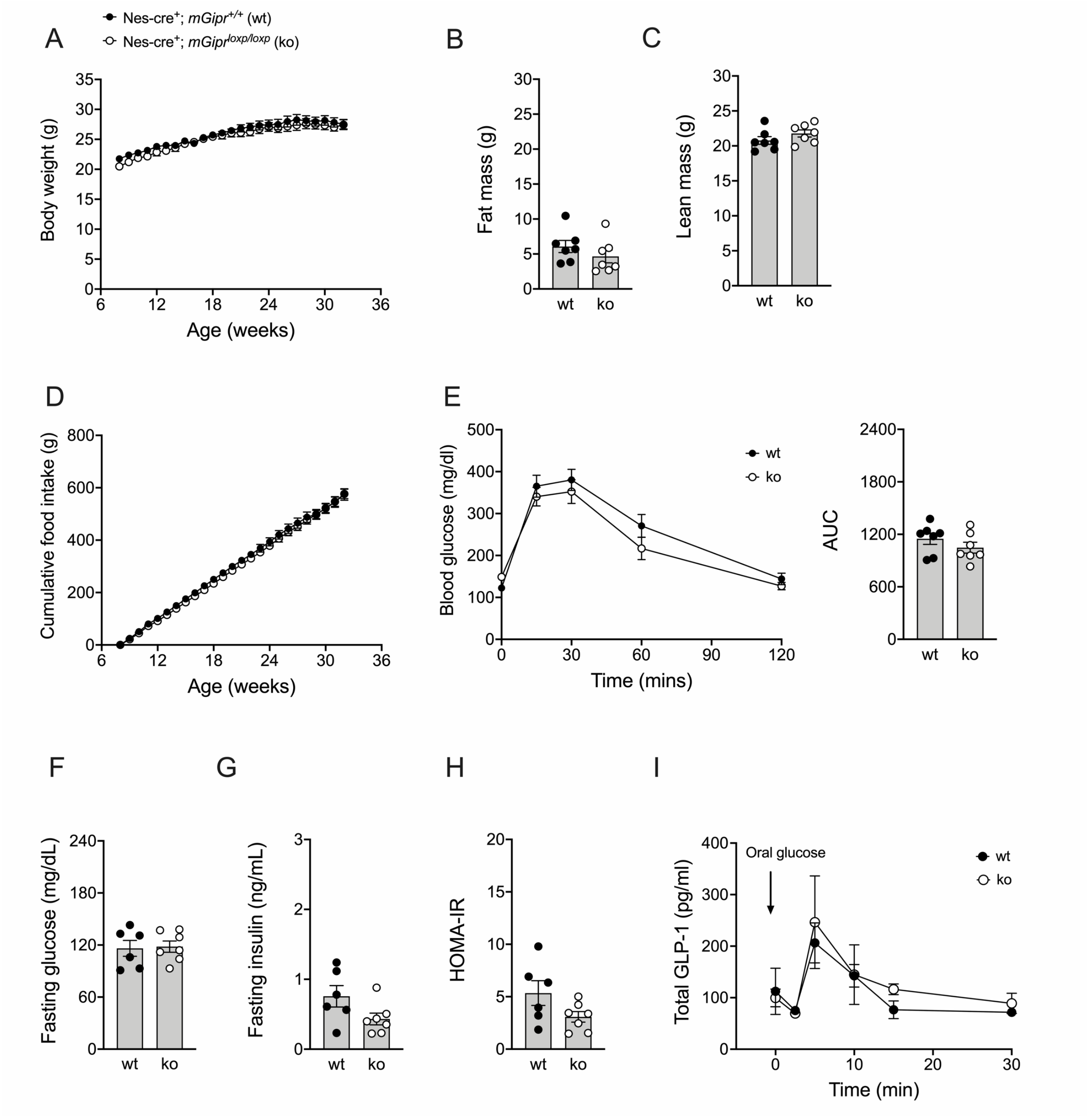

Suppl. Figure 2. Metabolic phenotype of Chow-fed CNS-*Gipr* ko mice. Body weight **(a)**, body composition at the age of 28 wks **(b,c)** and food intake **(d)** of male chow-fed C57Bl/6J wt and CNS-*Gipr* ko mice (N=7 each genotype). Food intake **(d)** was assessed per cage in double-housed mice (N=4 cages per genotype). Intraperitoneal glucose tolerance **(e)** in 28 wk old mice (N=7 each genotype) and fasting levels of blood glucose **(f)** and insulin **(g)** as well as HOMA-IR **(h)** in 32 wk old mice (N=6-7 each genotype). Oral glucose stimulation of GLP-1 secretion in 30 wk old mice (N=5-7 mice each genotype) **(i)**. Data represent means ± SEM. Asterisks indicate * $p<0.05$, ** $p<0.01$ and *** $p<0.001$. Longitudinal data **(a,d,e,i)** were analyzed using 2-way ANOVA with time and genotype as co-variables and Bonferroni post-hoc analysis for individual time-points. Bar graphs in panel (**b,c,e-h)** were analyzed using 2-tailed, 2-sided ttest.

Suppl. Figure 3 – Expression of *hGIPR* and assessment of body weight and blood glucose in *hGIPR* ko mice and different wt cohorts (Related to Figure 2).

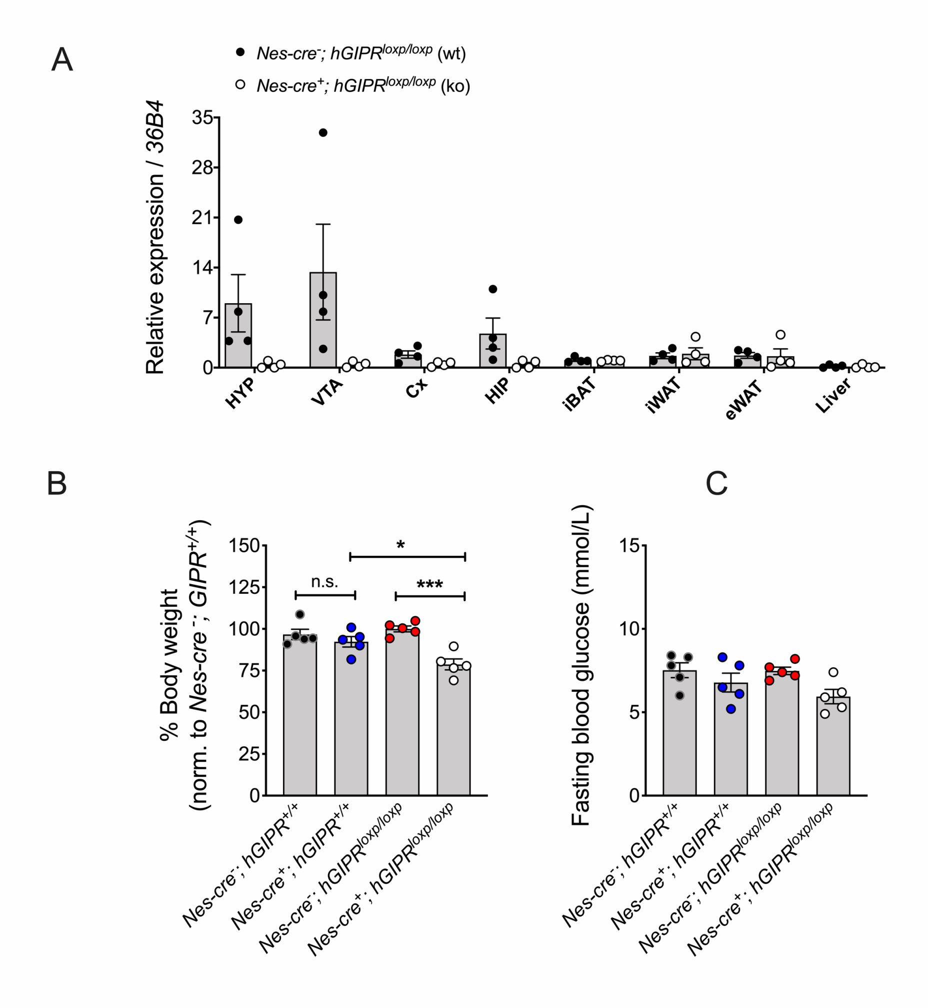

Suppl. Figure 3. GIPR ablation in CNS-*hGIPR* ko mice and assessment of body weight and blood glucose in different wt cohorts. Expression of h*GIPR* in hypothalamus, ventral tegmental area, cortex, hippocampus, inguinal brown adipose tissue (BAT), inguinal white adipose tissue (iBAT), epididymal white adipose tissue (eWAT) and liver of 12 wk old wt and CNS-*hGIPR* ko mice (N=4 each genotype) **(a)**. Difference in body weight **(b)** and fasting blood glucose **(c)** between nestin cre$^{-/-}$ *hGIPR*$^{+/+}$ mice, nestin cre$^{+/-}$ *hGIPR*$^{+/+}$ mice, nestin cre$^{-/-}$ *hGIPR*$^{flx/flx}$ mice and nestin cre$^{+/-}$ *hGIPR*$^{flx/flx}$ mice at the age of 12 wks (N=5 mice each genotype). Data represent means ± SEM. Asterisks indicate * $p<0.05$, ** $p<0.01$ and *** $p<0.001$. Bar graphs in (**a)** were analyzed using 2-tailed, 2-sided ttest, bar graphs in **(b,c)** were analyzed using 1-way ANOVA.

Suppl. Figure 4 – Central acyl-GIP effects on cFOS neuronal activity in the hypothalamus (Related to Figure 3).

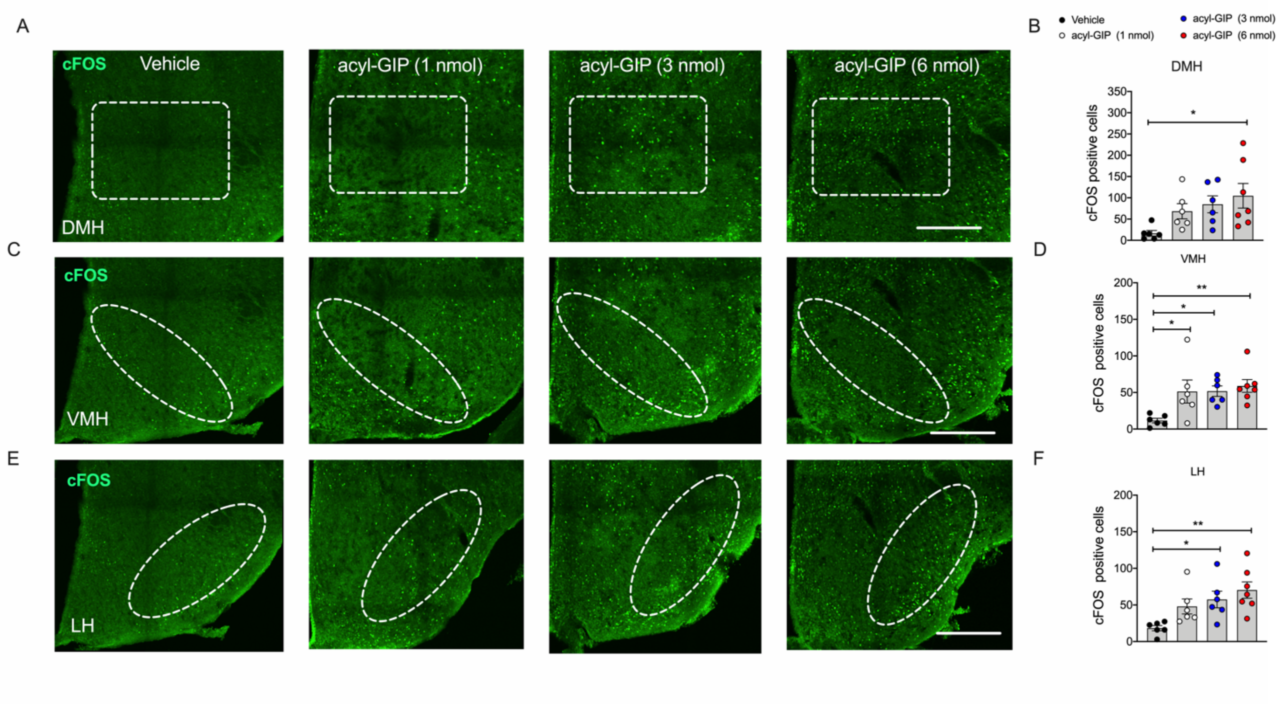

Suppl. Figure 4. Acyl-GIP effects on cFOS neuronal activity in the hypothalamus. cFOS neuronal activity in the dorsomedial nucleus **(a,b)**, the ventromedial nucleus **(c,d)** and lateral hypothalamus **(e,f)** following single acute icv treatment of DIO mice with 1, 3, or 6 nmol of acyl-GIP (N=6-7 mice each group). Data represent means ± SEM. Asterisks indicate * p<0.05, ** p<0.01 and *** p<0.001. Bar graphs in **(b,d,f)** were analyzed using 1-way ANOVA.

Suppl. Figure 5 – Effects of icv administered acyl-GIP on hypothalamic gene expression and lipid metabolism in DIO mice (Related to Figure 4).

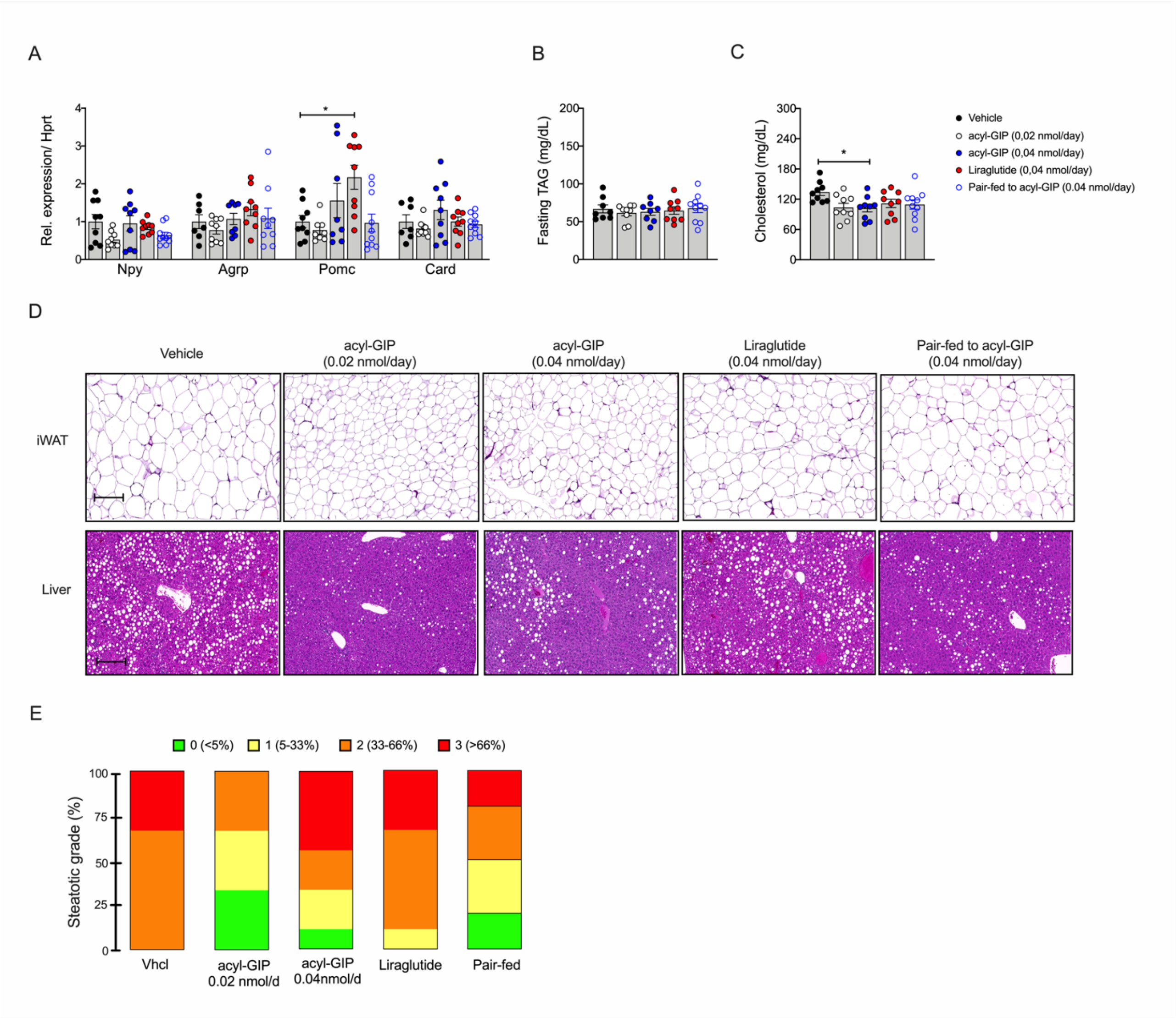

Suppl. Figure 5. Effects of icv administered acyl-GIP on hypothalamic gene expression and lipid metabolism in DIO mice. Effects of icv administered acyl-GIP (0.02 nmol/d) on hypothalamic expression of *neuropeptide y (Npy)*, *agouti-related protein (Agrp), proopiomelanocortin (Pomc) and cocaine- and amphetamine-regulated transcript (Cart)* in 32 wk old male DIO mice after 14 days of treatment (N=7-10 each group) **(a)**. Plasma levels of triglycerides **(b)** and cholesterol **(c)** after 14 days of treatment (N=8-10 mice each group). H&E staining of inguinal white adipose tissue (iWAT) and liver (scale bar 200 μm) (**d**) and quantification of the hepatic steatosis grade after 14 days of treatment (N=9-10 mice each group). Data represent means $\pm$ SEM. Asterisks indicate * $p<0.05$, ** $p<0.01$ and *** $p<0.001$. Bar graphs in **(a,b,c)** were analyzed using 1-way ANOVA.

Suppl. Figure 6 – Effects of acyl-GIP on thermogenic genes and BAT function (Related to Figure 5).

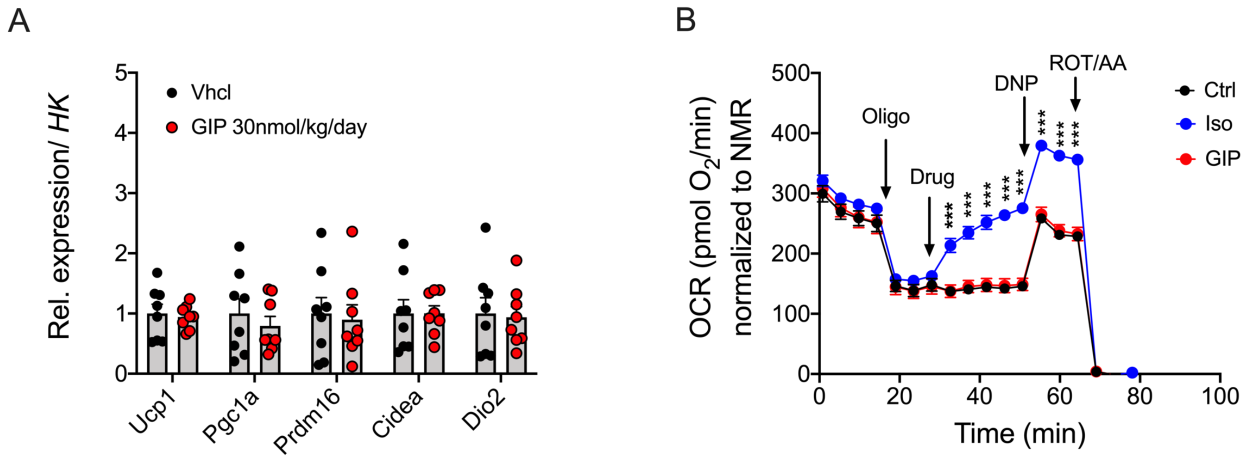

Suppl. Figure 6. Effects of acyl-GIP on thermogenic genes and BAT function. Expression of *Ucp1*, *Pgc1a*, *Prdm16*, *Cidea* and *Dio2* in BAT of male HFD-fed DIO mice treated daily with acyl-GIP (30 nmol/kg/day) for 7 days (N=8 mice each group) **(a).** Oxygen consumption rate (OCR) in individually differentiated (SV40 immortalized) brown adipocytes treated with oligomycin (5 µg/ml), test drug (isoproterenol,1 µM or GIP, 100 nM), dinitrophenol (DNP; 150 µM), followed by rotenone (5 µM) / antimycin A (2 µM) and 2DG (100 mM) **(b).** Drug effects were assessed in individually differentiated wells with N=6 Cntrl., N=3 isoproterenol, N=7 GIP. Data represent means ± SEM. Asterisks indicate * $p<0.05$, ** $p<0.01$ and *** $p<0.001$. Bar graphs in **(a)** were analyzed using 2-sided, 2-tailed ttest. Longitudinal data in (b) were analyzed using 2-way ANOVA with time and genotype as co-variables and Bonferroni post-hoc analysis for individual time-points.

Supplementary Table S1 – Additional Key Resource Data for used Oligonucleotides (Related to STAR Methods and Figures 1, 2, 4, Suppl. Figure 1, Suppl. Figure 3, Suppl. Figure 5, Suppl. Figure 6).

REAGENT or RESOURCE	SOURCE	IDENTIFIER
Oligonucleotides		
Pomc - F: 5'-CATTAGGCTTGGAGCAGGTC-3'	This paper	n/a
Pomc - R: 5'-TCTTGATGATGGCGTTCTTG-3'	This paper	n/a
Npy – F: 5'-TGGACTGACCCTCGCTCTAT-3'	This paper	n/a
Npy – R: 5'- TGTCTCAGGGCTGGATCTCT -3'	This paper	n/a
Agrp – F: 5'-CGGCCACGAACCTCTGTAG-3'	This paper	n/a
Agrp – R: 5'- CTCATCCCCTGCCTTTGC -3'	This paper	n/a
Cart – F: 5'-CGAGAAGAAGTACGGCCAAG-3'	This paper	n/a
Cart – R: 5'- GGAATATGGGAACCGAAGGT -3'	This paper	n/a
Ppib – F: 5'-GCATCTATGGTGAGCGCTTC-3'	This paper	n/a
Ppib – R: 5'- CTCCACCTTCCGTACCACAT -3'	This paper	n/a
Hprt – F: 5'-AAGCTTGCTGGTGAAAAGGA-3'	This paper	n/a
Hprt – R: 5'- TTGCGCTCATCTTAGGCTTT -3'	This paper	n/a
Gipr universal – F: 5'-CCAGGAGTGCCAAAAGATGTT-3'	This paper	n/a
Gipr universal – R: 5'-CCAGGAGTGCCAAAAGATGTT-3'	This paper	n/a
Gipr wt-specific – F: 5'-GTGTCCACGAGGTGGTGTTT -3'	This paper	n/a
Gipr universal – R: 5'-CCGACTGCACCTCTTTGTTG -3'	This paper	n/a
hGIPR– F: 5'-TGTTTGCTCCCGTGACAGAG -3'	This paper	n/a
hGIPR – R: 5'-CGACTGCACCTCCTTGTTGA -3'	This paper	n/a
Pomc– F: 5'-ACCCTCGTTTCTCTGCGCATC -3'	This paper	n/a
Pomc– R: 5'-AGGGCCCCTGAGCGACTGTA -3'	This paper	n/a
Npy– F: 5'- TGGACTGACCCTCGCTCTAT -3'	This paper	n/a
Npy– R: 5'- GGGGCGTTTTCTGTGCTTTC -3'	This paper	n/a
AgRP– F: 5'- GCCATCTTCCACCTTTGCAG -3'	This paper	n/a
AgRP– R: 5'- CCTGGTCAGGCCTTCTGATG -3'	This paper	n/a
Cart– F: 5'- CCTGCTGCTACTGCTACCTTT -3'	This paper	n/a
Cart– R: 5'- CGCTTCGATCAGCTCCTTCT -3'	This paper	n/a
Bdnf– F: 5'- CATCCGAGGACAAGGTGGCTTG -3'	This paper	n/a
Bdnf– R: 5'- GCCGAACTTTCTGGTCCTCATC -3'	This paper	n/a
36B4– F: 5'- GCCGTGATGCCCAGGGAAGACA -3'	This paper	n/a
36B4– R: 5'- AACCCGCGGCCTTCTACGAG -3'	This paper	n/a
Ucp1– F: 5'- GGCCTCTACGACTCAGTCCA-3'	This paper	n/a
Ucp1– R: 5'-TAAGCCGGCTGAGATCTTGT-3'	This paper	n/a
Prdm16– F: 5'- CCGCTGTGATGAGTGTGATG-3'	This paper	n/a
Prdm16– R: 5'- GGACGATCATGTGTTGCTCC-3'	This paper	n/a
Cidea– F: 5'- AATGGACACCGGGTAGTAAGT-3'	This paper	n/a
Cidea– R: 5'- CAGCCTGTATAGGTCGAAGGT-3'	This paper	n/a
Dio2– F: 5'- TGCCACCTTCTTGACTTTGC-3'	This paper	n/a
Dio2– R: 5'- GGTTCCGGTGCTTCTTAACC-3'	This paper	n/a
Pgc1a– F: 5'- AGCCGTGACCACTGACAACGAG-3'	This paper	n/a
Pgc1a– R: 5'- GCTGCATGGTTCTGAGTGCTAAG -3'	This paper	n/a
